# A comprehensive analysis of humanized mouse models for the study of cancer immunotherapies

**DOI:** 10.3389/fimmu.2026.1730378

**Published:** 2026-04-07

**Authors:** Philippe De La Rochere, Laure Loumagne, Melanie Rathaux, Marine Dubois, Jordan Denizeau, Fariba Nemati, Sophie Viel, Dario Rocha, Tamara Slavnic, Jayant Thatte, Henry Qixiang Li, Xuesong Ouyang, Christine Sedlik, Didier Decaudin, Georges Azar, Sukhvinder Sidhu, Eliane Piaggio

**Affiliations:** 1 Institut national de la santé et de la recherche médicale (INSERM) U932, and SIRIC Translational Immunotherapy Team, Translational Research Department, Institut Curie, PSL Research University, Paris, France; 2 Sanofi Oncology, Vitry Sur Seine, France; 3 Laboratory of Preclinical Investigation, Translational Research Department, Institut Curie, PSL Research University, Paris, France; 4 IT&M STATS, Groupe IT&M, Neuilly-sur-Seine, France; 5 Crown Bioscience Inc., San Diego, CA, United States; 6 Department of Medical Oncology, Institut Curie, Paris, France

**Keywords:** cancer, humanized mice, immune checkpoints, immunodeficient mice, immunotherapy

## Abstract

**Introduction:**

Humanized immune system (HIS) mouse models, generated by engrafting tumors and hematopoietic cells of human (Hu) origin into immunodeficient host mice, effectively recapitulate key aspects of the crosstalk between human immune cells and tumors. These models represent a valuable tool for the preclinical evaluation of immunotherapies.

**Methods:**

In this study, we provide a comprehensive comparison of two widely used HIS models: the Hu-CD34+ model, which engrafts Hu-hematopoietic cells derived from Hu-CD34+ hematopoietic stem cells (HSCs), and the Hu-PBMC model, which utilizes Hu-peripheral blood mononuclear cells (PBMCs).

**Results:**

We assess the kinetics, quality and extent of immune cell engraftment, as well as the development of graft-versus-host disease (GVHD). Additionally, we investigate the impact of different immunodeficient host mouse strains on immune cell reconstitution in the Hu-CD34+ model. Both HIS models were engrafted with human tumors derived from either cell lines or patient-derived xenografts (PDX), revealing distinct immune-tumor interactions that influenced antitumor responses. Notably, tumor responses to T-cell-directed therapies, including anti-PD1 antibodies, IL-2-anti-IL-2 antibody complexes, and T-cell engagers, varied across these models.

**Discussion:**

Our findings provide novel insights into the properties and limitations of HIS models, offering a critical resource for optimizing next-generation immuno-oncology strategies and guiding the design of future therapeutic interventions.

## 1 Introduction

The blockade of immune checkpoints with antibodies (Ab) anti-CTLA-4, anti-PD1 and anti-PD-L1, has given impressive clinical results and manageable safety profiles, positioning immunotherapy as a promising therapeutic strategy to treat cancer (1, 2). However, a significant roadblock to develop novel immunotherapies is the paucity of translationally relevant *in vitro* and *in vivo* models. Therefore, there is a significant and urgent need to develop *in vivo* mouse tumor models to help evaluate the mechanism of action of these novel immunotherapies, identify biomarkers of response and toxicity, and prioritize combinatorial approaches of immunotherapies (such as bi-specific Abs, cytokines & vaccination) with targeted therapies, radiotherapy, or chemotherapy.

The major approaches used to assess cancer immunotherapies in preclinical studies today include syngeneic mouse tumor models and genetically engineered mouse models both in fully immune-competent hosts, and humanized immune system (HIS) models in immunodeficient mice (3). While the first two approaches have been intensively used to bridge cancer biology and translational research, one major inconvenience is that they rely on the mouse immune system that does not fully recapitulate its human counterpart (4). Moreover, these models do not allow either the evaluation of therapeutic agents that are not cross-reactive to mouse, or the direct study of human immune cell populations. Therefore, HIS mouse models, that can reconstitute a functional human immune system and can be engrafted with human tumors represent a valuable tool to evaluate immunomodulatory compounds in a more physiologically relevant setting (5, 6).

In HIS models, immunodeficient host mice are engrafted with human hematopoietic cells and tumors of human origin (5, 6). In general, two different sources of hematopoietic cells are used: CD34^+^ HSC (hematopoietic stem cells) (Hu-CD34^+^ model) or peripheral blood mononuclear cells (PBMCs) (Hu-PBMC model). The HSC approach is based on the injection of CD34^+^ progenitor cells that have the capacity of self-renewal and long-term reconstitution of several human immune cell subtypes, including T cells, B cells, NK cells, DC, monocytes and neutrophils. However, upon reconstitution, not all human cell subtypes are completely functional. More recently, several strains of highly immunodeficient mice were engineered to overcome this issue, including the NSG (7), NSG‐SGM3 (8), NOG‐EXL (9), BRGS (10) and BRGSF (11) strains, but to our knowledge, comprehensive side-by-side comparisons of immune cell reconstitution in these mouse models are scarce (12).

In the Hu-PBMC model, PBMCs are obtained from healthy donor (HD) PBMCs, and T cells are the predominant population that repopulates the host mouse. The major limitation of this model is fatal acute xeno-graft-versus-host-disease (GvHD) (13) that occurs 3–5 weeks after PBMC injection, restricting the evaluation of anti-tumor T cell responses to a relatively short period of time.

The main hurdles of HIS mice are: i) the presence of murine innate immune cells, such as macrophages, dendritic cells and neutrophils, that negatively influence the engraftment of human immune cells, ii) the difficulty to obtain matched donor and tumor samples, iii) the variations in the immune reconstitution kinetics, iv) the incomplete reconstitution of human immune cell subsets, v) the development of xeno-GvHD, all of which influence therapeutic outcomes; and vi) the high cost. Understanding these variables is critical for translational research because they determine the predictive value of HIS models for clinical responses and toxicities.

In this study, we systematically compare CD34^+^ hematopoietic stem cell–based and PBMC-based HIS models across multiple immunodeficient strains, assessing immune reconstitution, donor variability, tumor responsiveness, and efficacy of T-cell–directed therapies including PD-1 blockade, IL-2/anti–IL-2 complexes, and bispecific T-cell engagers. Our findings provide guidance for model selection: identifying conditions that favor checkpoint inhibitor evaluation versus T-cell engager testing, defining donor-dependent variability, and establishing dosing strategies to balance immune reconstitution and GvHD onset. These insights enhance the translational relevance of HIS models and inform the design of preclinical studies aimed at optimizing immuno-oncology strategies and developing patient-tailored therapies.

## 2 Materials and methods

### 2.1 Mice

NOD.Cg-*Prkdc^scid^ Il2rg^tm1Wjl^*/SzJ (NSG) and NOD.Cg-*Prkdc^scid^ Il2rg^tm1Wjl^* Tg(CMV-IL3,CSF2,KITLG)1Eav/MloySzJ (NSG-SGM3) were purchased from The Jackson Laboratory; NOD.Cg-*Prkdc^scid^ Il2rg^tm1Sug^*/JicTac (NOG) and NOD.Cg-*Prkdcscid Il2rgtm1Sug* Tg(SV40/HTLV-IL3,CSF2)10-7Jic/JicTac (NOG-EXL) were purchased from Taconic Biosciences. Experiments were performed in Sanofi-, Institut Curie- and Crown Bioscience- mouse facilities. Animal studies were conducted at Sanofi’s Vitry-sur-Seine research facility In environmental conditions including room temperature (22 °C ± 2 °C), relative humidity (55% ± 15%) and lighting times (12-hour light/dark cycle). In Institut Curie, mice were housed in a non-barrier facility and enrolled in experiments at 6–10 weeks of age. Mice were co-housed under standardized conditions with ambient temperature maintained between 20 and 24 °C, an average relative humidity of 40–70% and a 12 h light/12 h dark cycle.

Animals were group-housed in European standard housing with appropriate environmental enrichment and were provided with standard laboratory diet and water ad libitum. All animals were acclimated to the facility for at least 1 week prior to study initiation. Animal welfare was monitored daily by trained animal care staff.

In Crown Bioscience, mice were housed in SPF facility and enrolled in experiments at 6–10 weeks of age. Mice were co-housed under standardized conditions with ambient temperature maintained between 20 and 26 °C, an average relative humidity of 30–70% and a 12 h light/12 h dark cycle.

### 2.2 Human immune cells

Hu-PBMCs were isolated from buffy coats provided by the Etablissement Français du Sang (EFS), after written informed consent. Hu-CD34^+^ cells from cord blood were obtained from ABCell-Bio (Evry, France).

### 2.3 Hu-CD34^+^ HSC- mice

Humanization was performed according to mice provider instructions depending on the mouse strain at Sanofi mouse facilities. Briefly, mice received sub-lethal total body irradiation from an X-ray source (CP 160 Faxitron X-ray): 1.4Gy for 3-week-old NSG, 1.1Gy for 3-4-week-old NOG, 1 Gy for 5-week-old NSG-SGM3, and 0.6 Gy for 4-6-week-old NOG mice. Eighteen to 24 hours post-irradiation, mice were injected intravenously (iv) with human CD34^+^ HSC cells (1.3x10^5^ cells for NSG and NOG, 1x10^5^ for NSG-SGM3, and 0.5x10^5^ for NOG-EXL mice). Mice humanization at Crown Bioscience were performed using CD34^+^ HSC–humanized NSG mice obtained from The Jackson Laboratory (Bar Harbor, ME, USA) and only mice reconstituted with > 25% Hu-CD45^+^ cells in blood by 12 weeks post-HSC inoculation are used.

### 2.4 Hu-PBMC-NSG mice

NSG mice were grafted with human tumor cell lines (CDX) or patient-derived xenografts (PDX) and then iv injected with various doses of Hu-PBMCs. In each experiment, Hu-PBMCs from 2 independent donors - unless otherwise explained- were administered in separate mice to account for donor variability. However, identical donors were not systematically matched across all tumor models (PDX vs CDX) due to practical constraints in donor availability and experimental timelines. Hu-PBMCs were isolated using Ficoll gradient (lymphoprep, Stemcell).

### 2.5 Determination of GvHD onset

GvHD onset was defined as sustained body weight loss >20%, which is a commonly used criterion in Hu-PBMC models (14). A limitation of this study is that we only used body weight loss as a common parameter across the different participating laboratories, although inclusion of other parameters as posture changes, fur condition, skin lesions or welfare scoring systems, would have improved GVHD definition (15).

### 2.6 Tumor cell lines and patient-derived xenografts

Human triple negative breast cancer MDA-MB231 cell line, NSCLC cancer HCC827 cell line (ATCC) and HT-29 colorectal cell line (ATCC) were grown in RPMI medium (Life Technologies) supplemented with 10% heat-inactivated fetal bovine serum (Biosera), glutamine and penicillin-streptomycin (Life Technologies). 3x10^6^ HT-29 or 5x10^6^ MDA-MB231 or HCC827 tumor cells were injected subcutaneously (sc) in immunodeficient mice and tumor growth was measured using a metric caliper twice a week.

PDXs from breast or lung origin were obtained following informed consent from patients undergoing surgery. For humanization experiments, tumor fragments from PDX were grafted into the interscapular fat pad. For the mini-PDX trial using SCLC-derived PDXs, tumors from PDX were dissociated using Collagenase/DNase into single cell suspension and 100-200x10^3^ cells were mixed with Cultrex matrix at 1:1 ratio and implanted sc. PDX models are available from D. Decaudin, the EuroPDX Data Portal (https://edirex-dataportal.ics.muni.cz/), and from Crown Bioscience (https://www.crownbio.com). The BC138 PDX, (also referred to as HBCx-8 (16)) was established from a 78-year-old patient diagnosed with triple-negative breast cancer whith no prior treatment (N0, M0 at the time of surgery). Lung cancer PDX models were derived from patients with a mean age of 63 years (range: 50 to 77) including 8 NSCLC patients (7 untreated (LCF13, LCF15, LCF16, LCF25, LCF26, LCF29, LU5173) and 1 neoadjuvant chemotherapy treated (LCF17), and 7 SCLC patients (1 untreated (LCF22) and 6 without retrievable information for treatment (LU5203, LU5220, LU5223, LU5249, LU5250, LU5255). No patient received immunotherapy.

### 2.7 *In vivo* treatments

Anti-PD1 mAbs (Nivolumab (Opdivo^®^, BMS) or Pembrolizumab (Keytruda^®^, MDS) were injected intra-peritoneally (ip), biweekly, starting 3 to 10 days after PBMCs injection, at 10mg/Kg. Control mice were untreated or received human IgG4 isotype antibody as mentioned in legends. IL-2 based immunotherapy using IL-2Cxs was prepared as described (17) and IL-2Cxs were injected ip for five consecutive days, 5 days after PBMCs injection and biweekly in the weeks after. EpCAM-CD3 Ab was injected iv at 2.5mg/kg biweekly.

### 2.8 Tissue processing and flow cytometry analysis

Tumors, spleen and bone marrow (BM) were collected in PBS-2% FBS. Femurs and tibias were flushed and collected BM was dissociated through a cell strainer. When necessary (spleen, BM), red blood cells (RBC) were lysed twice with Fixative-Free Lysing Solution (Invitrogen) and used for flow cytometry staining. Peripheral blood (PB) was collected in EDTA or heparin coated tubes and cells were stained prior RBC lysis.

Tumor fragments were digested with DNAse (10mg/ml, Roche), and liberase LT (5mg/ml, Roche), processed on a Gentlemacs dissociator (Miltenyi Biotec), filtered through a 100µm cell strainer (BD Biosciences), washed with PBS.

Cell suspensions were used for FACS staining and first incubated with antibodies to block human and mouse Fcγ receptors (-Human TruStain FcX™ from Biolegend and rat anti-mouse CD16/32 from BD Biosciences); and then stained for surface markers. Antibodies and viability dye are listed in **Supplementary Table 1**. Fortessa cytometer (BD Biosciences) was used for cell acquisition and FlowJo software v10.0.8 (TreeStar) for data analysis.

### 2.8 Plasma cytokine measurement

EDTA-plasma samples were analyzed using the MSD U-PLEX assay platform (U-PLEX Proinflam Combo 1 Human K15049K; Mesoscale Discovery) according to manufacturer’s instruction. Plates were analyzed in a MSD Discovery Workbench.

### 2.9 Statistical analyses

Statistical analyses used are detailed in the figure legends and significance set at p < 0.05.

For tumor volume data, a Two-Way Analysis of Variance Type (ANOVA Type) was performed on the changes from baseline when baseline was available, and on raw tumor volumes otherwise with time as a repeated factor. For cytokine concentration and percentage of immune cells measured at week 18 only, a One-Way ANOVA was performed on log-transformed data with time as a repeated factor for cytokines and with a random effect on donors for % cells. Frequency of cells were also analyzed with non-parametric t test on raw or log transformed data when appropriate. For all models, adjustment for multiplicity was applied when multiple comparisons were performed. Analyses were performed using SAS^®^ version 9.4 for Windows 7 and with GraphPad Prism v10 and graphs were generated with GraphPad Prism v7 and v10.

## 3 Results

### 3.1 Characterization of the Hu-CD34^+^ model: immune cell reconstitution and GvHD development

Although different mouse strains have been used for immune evaluation, few studies have directly compared immune cell reconstitution in these mouse models (Maser 2020).

To address this gap, we conducted a side-by-side comparison of the four most commonly used genetically modified immunodeficient mice: NSG, NSG-SGM3, NOG, and NOG-EXL. These strains differ in their ability to support human immune cell development, particularly in the myeloid versus lymphoid compartments. The NSG and NOG mice differ in their genetic modifications, but both achieve a functional knockdown of the IL2R gamma chain, resulting in a profound immunodeficiency with absent T, B, and NK cells. The NSG-SGM3 and NOG-EXL strains are transgenic for the expression of human IL-3 and GM-CSF for both strains and KIT-ligand for NSG-SGM3 only, which enhances the differentiation and survival of human myeloid cells.

**Figure 1:**
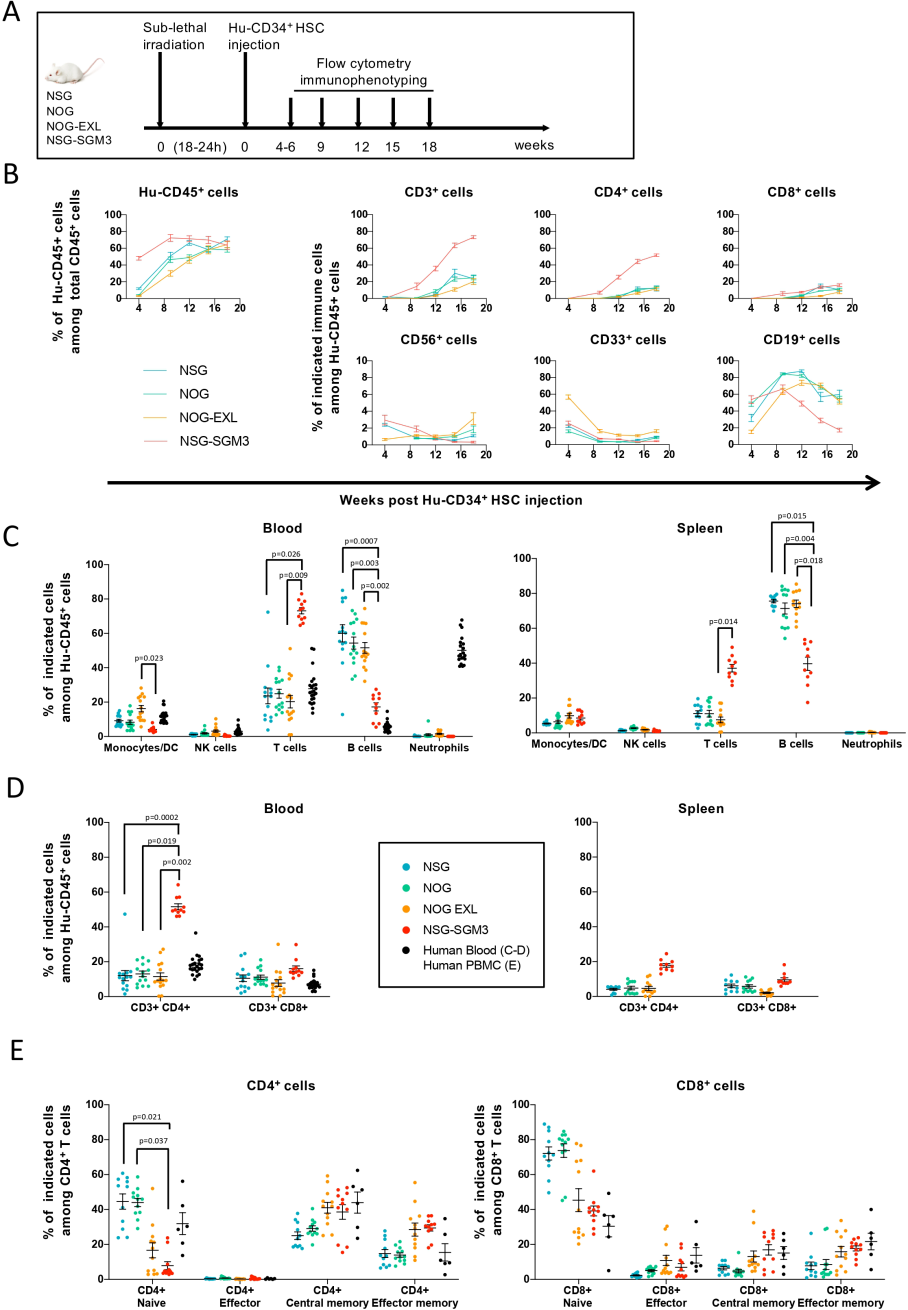
Human immune cell reconstitution upon Hu-CD34^+^ HSC injection in different strains of immunodeficient mice. **(A)** Experimental design. 50-130x10^3^ Hu-CD34^+^ HSC were injected into NSG, NOG, NOG-EXL or NSG-SGM3 mice. Blood was recovered at weeks 4 to 6, 9, 12, 15 and 18 post-injection of Hu-CD34^+^ HSC to quantify human reconstitution by flow cytometry analysis. **(B)** Frequencies (%) of Hu-CD45^+^ cells population relative to total CD45^+^ cells (Hu-CD45^+^ + m-CD45^+^) and subpopulations among Hu-CD45^+^ cells, in blood over time. **(C, D)** Distribution of the Hu-CD45^+^ cell subpopulations relative to Hu-CD45^+^ cells in blood and spleen of different mouse strains. Mice were sacrificed 18 weeks after Hu-CD34^+^ HSC injection and the distribution of the immune cell populations was determined by flow cytometry. Monocytes/DC are defined as Non neutrophils CD33^+^ cells, NK cells as CD3^-^ CD56^+^ cells, T cells as CD3^+^ CD56^-^ cells, B cells as CD19^+^ cells and Neutrophils as SSC^high^ CD16^high^ cells as described in the gating strategy (**Supplementary Figure 1A**). **(E)** T cell differentiation state in Hu-CD34^+^-humanized mice. Frequencies (%) of naïve, memory, effector memory, and effector cells relative to CD4^+^ T cells (left panel) and CD8^+^ T cells (right panel) were determined by flow cytometry analysis. Gating strategy is similar to the one used for Hu-PBMC-NSG mouse as illustrated in **Supplementary Figure 2C**. Data were obtained from mice reconstituted with 2 or 3 different Hu-CD34+ HSC donors with n=10–15 mice per mouse strain for blood and spleen data. Data is expressed as individual dots and mean ± SD. p values were obtained with a One-Way Analysis of Variance on log-transformed data and are adjusted with Tukey’s correction for multiplicity. Absence of reported p-values indicates non-significant results (p ≥ 0.05). These experiments were performed at Sanofi.

To assess engraftment, instead of using absolute immune cell numbers, we employed the percentage of human chimerism -defined as the proportion of Hu-CD45^+^ cells among total CD45^+^ cells in blood, spleen, and bone marrow—as a surrogate for engraftment efficiency in humanized mouse models (5, 18). This metric is widely adopted because it reflects the degree of human immune system reconstitution and correlates with functional immune responses. Following humanization, all strains showed successful engraftment of human hematopoietic cells, as indicated by sustained Hu-CD45+ cell presence in blood up to 18 weeks post-HSC injection (**Figure 1**, **Supplementary Figures 1A–E**). Although the overall kinetics of Hu-CD45+ reconstitution was similar across strains, NSG-SGM3 mice exhibited an accelerated immune reconstitution with significantly higher Hu-CD45+ percentages at earlier time points. By week 18 post-humanization, Hu-CD45+ immune cells constituted approximately 50–70% of the total hematopoietic cells in blood across all strains.

Distinct immune cell subsets displayed different engraftment kinetics. NK (CD56+), non-neutrophil myeloid cells (CD33+) and B cells (CD19+) were detectable as early as week 4 post-humanization, whereas T cells (CD3+) were not observed until week 12 in most strains, except for NSG-SGM3, which showed earlier and higher T-cell reconstitution, particularly CD4+ T cells (**Figure 1B**). Notably, in NSG-SGM3 mice, B-cell reconstitution was significantly lower than in the other strains.

At week 18 post-HSC injection, immune cell distribution in blood and spleen was assessed relative to human donor blood. T, NK, and non-neutrophil myeloid cells (monocytes/DC) were present in similar proportions to those in healthy donors, while B cells were overrepresented in all strains except NSG-SGM3 (**Figure 1C**). Neutrophils remained absent in circulation across all models, and despite enhancements in myeloid differentiation in NSG-SGM3 and NOG-EXL strains, no significant improvements in myeloid reconstitution were observed at this time point (**Figure 1C**, **Supplementary Figures 1E–G**). Interestingly, human neutrophils were detected in the bone marrow of all strains, albeit at significantly lower frequencies in NSG-SGM3 mice (**Supplementary Figure 1F**).

Analysis of T-cell subsets revealed that NSG-SGM3 mice had a significantly higher proportion of CD4+ T cells, and a reduced proportion of B cells compared to the other strains (**Figure 1D**). Moreover, the overall T-cell compartment in all models exhibited normal frequencies of naïve, effector, central memory, and effector memory cells, although NSG-SGM3 mice displayed a relative depletion of naïve T cells (**Figure 1E**). In contrast to human donor blood, NK cells in all models lacked the characteristic predominance of cytotoxic NK cells over cytokine-producing NK cells (**Supplementary Figures 1B, C**).

Concerning GVHD development, 7 out of 18 NSG-SGM3 humanized mice (39%) had to be euthanized due to severe weight loss between weeks 10 and 17 post-humanization (**Supplementary Figure 1H**), suggesting potential toxicity concerns in this strain. Although not directly assessed in this manuscript, several publications have reported that Hu-NSG-SGM3 have decreased lifespan compared with NSG/NOG and NOG-EXL due to myeloid hyperactivation (12, 19, 20). However, none of the humanized NSG, NOG, or NOG-EXL mice displayed clinical signs of GvHD throughout the study period, supporting their suitability for long-term immunotherapy evaluation.

### 3.2 Characterization of the Hu-PBMC model: immune cell reconstitution and GvHD development

The Hu-PBMC-NSG model is widely used to study human immune responses, but a key limitation is the inevitable onset of GvHD, which occurs as human T cells recognize and attack murine tissues (21, 22). Therefore, we sought to define an optimal therapeutic window between immune reconstitution and overt GvHD onset.

**Figure 2:**
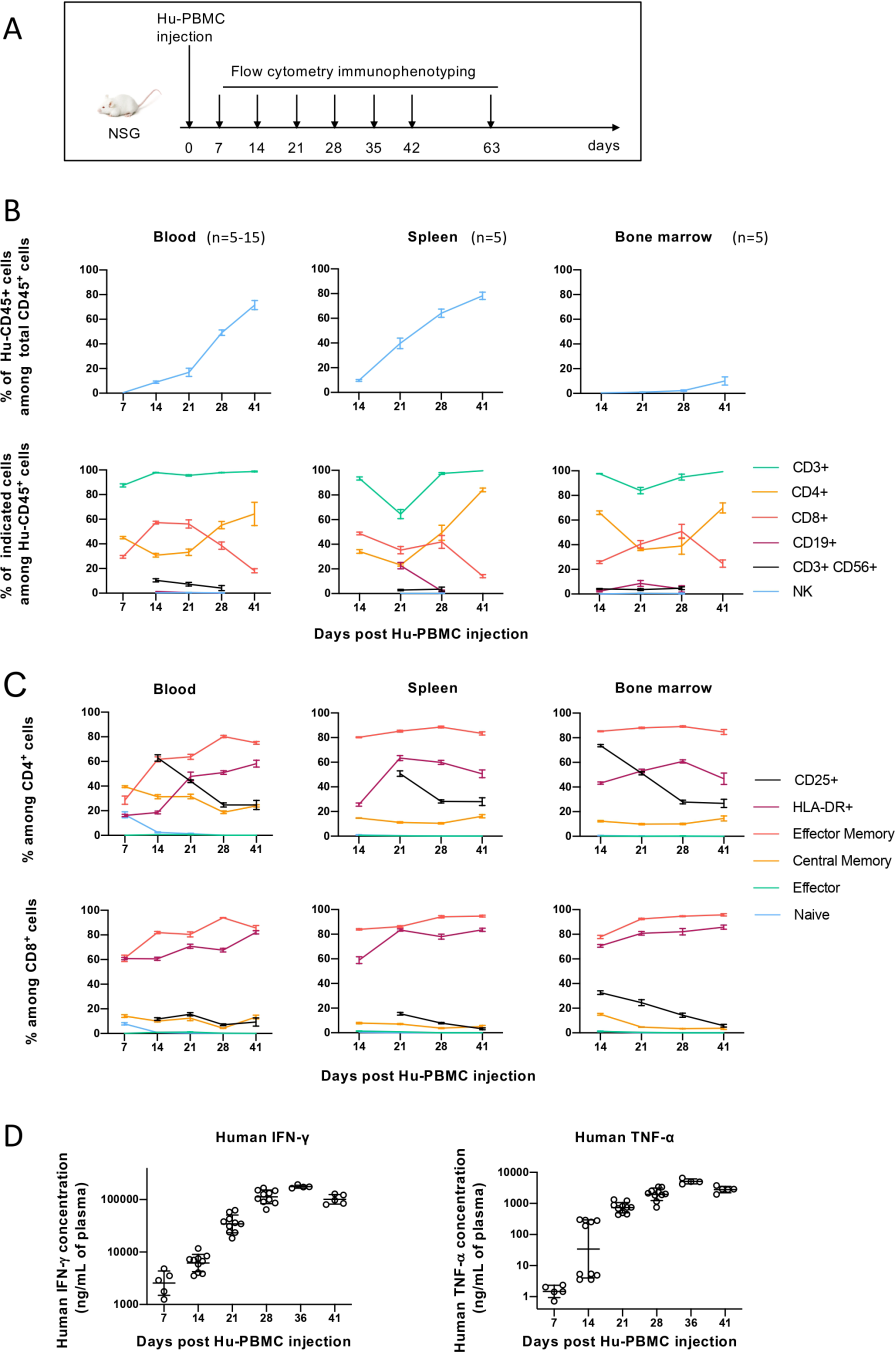
Human immune cell reconstitution upon Hu-PBMCs injection in NSG mice. **(A)** Experimental design. Non-irradiated NSG mice from 8 to 14 weeks of age were injected with 10x10^6^ of Hu-PBMCs. Blood, spleen and bone marrow were recovered at illustrated time points to quantify human reconstitution by flow cytometry analysis. **(B)** Changes in Hu-CD45^+^ cells reconstitution in blood (n=5-10), spleen (n=5) and bone marrow (n=5). Frequencies (%) of Hu-CD45^+^ cells relative to total CD45^+^ cells (Hu-CD45^+^ + m-CD45^+^) (upper panel) and of human immune cells subpopulations (CD3^+^ T cells, CD3^+^ CD4^+^ T cells, CD3^+^ CD8^+^ T cells, CD56^+^ CD3^+^ cells, CD19^+^ B cells and NK cells) relative to Hu-CD45^+^ cells (lower panel) at different time points. The gating strategy is described in **Supplementary Figure 1A**. Data is expressed as mean ± SD of representative results obtained with one out of five donors. **(C)** Changes in differentiation and activation state of CD4^+^ (upper panel) and CD8^+^ T cells (lower panel) in blood (n=5-15), spleen (n=5) and bone marrow (n=5) of reconstituted NSG mice. Frequencies (%) of effector memory, central memory, effector and naïve cells as well as activation marker-expressing cells (CD25 and HLA-DR) relative to CD4^+^ or CD8^+^ T cells were determined by flow cytometry analysis at different time points. The gating strategy is described in **Supplementary Figure 2C**. Data is expressed as mean ± SD from one representative experiment out of five. **(D)** From this representative experiment are shown the plasma concentrations (individual dots and geometric mean multiplied or divided by geometric SD) of human IFN-γ and TNF-α in Hu-PBMC-NSG mice at different time points. p<0,05 for both cytokines until day 28. p values were not represented for clarity and were obtained with One-Way ANOVA on log data and are adjusted with Bonferroni Holm’s correction for multiplicity. These experiments were performed at Sanofi.

To optimize PBMC dosing for delayed GvHD onset, we injected non-irradiated NSG mice with varying PBMC doses and monitored immune engraftment and GvHD development (**Figure 2A**, **Supplementary Figures 2A, B**). Mice were considered stably reconstituted when at least 2% of Hu-CD45+ cells were detected in blood at two different days. GvHD onset was defined when weight loss was >20%.

Our results indicate that: i) A minimum dose of 5 × 10^6^ CD3+ T cell-containing PBMCs is required for robust immune reconstitution in most mice. ii) The maximal level of reconstitution (% Hu-CD45+ in blood) is positively correlated with the PBMC dose. iii) The frequency of mice developing GvHD is positively correlated with the number of PBMCs present in the inoculum. iv) The timing of GvHD onset correlates with PBMC dose, with clinical symptoms appearing earlier in mice receiving higher PBMC numbers. And v) GvHD symptoms typically emerge when Hu-PBMC levels in blood exceed 30%, though lower thresholds were occasionally observed.

Based on these findings, we determined that injecting 5-10 × 10^6^ CD3+ T cell-containing PBMCs provided the best balance between immune reconstitution and manageable GvHD onset (**Supplementary Figures 2B, C**) and these doses were used for subsequent experiments. Additionally, to account for inter-donor variability, subsequent experiments incorporated PBMCs from at least two different donors per experimental group.

Kinetic analysis of immune reconstitution (**Figure 2B**) revealed that Hu-CD45+ cells appeared in circulation as early as day 7–14 post-injection, reaching peak levels (~70% in blood, ~80% in spleen, ~10% in bone marrow) by day 41. CD3+ T cells comprised ~90% of engrafted Hu-CD45+ cells by day 7. Initially, CD4+ and CD8+ T cells were present in equal proportions, but from day 28 onward, CD4+ T cells became dominant. A similar trend was observed in the spleen and in bone marrow. From day 7 to day 41 post-PBMCs injection, only a minor proportion of CD3+ CD56+ cells were found in the blood and in the spleen and few B cells were found in the bone marrow (**Figure 2B**). Results are representative from 4 experiments with different PBMC donors, with only CD4/CD8 ratio being variable according to the donor (data not shown).

We analyzed the dynamics of T cell differentiation and activation in Hu-PBMC-NSG mice, comparing them to PBMCs from healthy donors (**Figure 2C**, **Supplementary Figures 2D, E**). T cells rapidly acquired an activated phenotype, with HLA-DR expression increasing from day 7 onward. CD4+ central memory T cells declined over time, while effector memory T cells expanded, and CD25 expression peaked at day 14 before decreasing. CD8+ T cells showed even earlier activation, with ~60% becoming effector memory cells by day 7, expressing HLA-DR, and increasing further over time. Central memory and CD25+ cells represented 15% of CD8+ T cells and were stable from day 14. Naïve and terminal effector CD4+ and CD8+ T cells were barely detectable beyond day 14. Similar trends were observed in the spleen and bone marrow. These changes correlated with rising levels of IFN-γ and TNF-α in blood, highlighting sustained immune activation and pro-inflammatory cytokine production in the model (**Figure 2D**).

Collectively, our data highlight the rapid and robust T-cell engraftment in Hu-PBMC-NSG mice but also confirm the inevitability of GvHD onset. Nevertheless, by optimizing PBMC dosing, we identified a 2–4-week therapeutic window during which T-cell-targeting immunotherapies can be evaluated without overt GvHD.

### 3.3 Pre-clinical evaluation of anti-PD1 mAb treatment in Hu-CD34^+^ mice engrafted with patient-derived xenografts or with tumor cell lines (cell line-derived xenograft)

Hu-CD34+ humanized mice offer several advantages over Hu-PBMC mice, including their superior reconstitution of multiple immune cell types, reduced T-cell activation, and minimal to no GvHD. These characteristics enable extended immunotherapy studies that are not feasible in the Hu-PBMC model. Here, we tested the ability of Hu-CD34+ mice to mount anti-tumor immune responses against PDXs or tumor cell lines following immune checkpoint inhibition (ICI) therapy.

**Figure 3:**
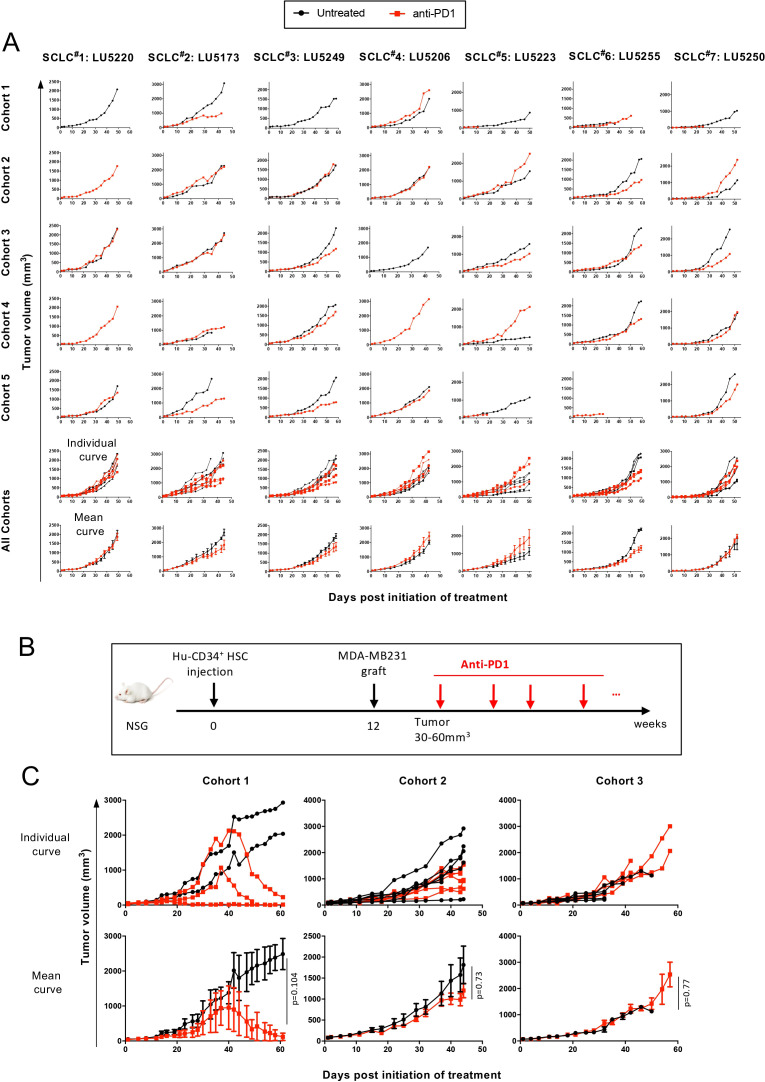
Effect of anti-PD1 mAb treatment on PDXs or cell line tumor growth in a Hu-CD34^+^-NSG mice. **(A)** A N of 1 mini-PDX trial study was performed in NSG mice which were inoculated with 100x10^3^ Hu-CD34^+^ HSCs from 5 different donors. Mice reconstituted with > 25% Hu-CD45^+^ cells in blood by 12 weeks post-HSC inoculation are used. The 5 Hu-CD34^+^ cohorts were engrafted with 7 models of SCLC PDXs and for each cohort/PDX combination, one mouse was injected with isotype control antibody (named “untreated”, black lines) or anti-PD1 mAb, Pembrolizumab (red lines). Tumor growth curves of individual mouse are shown and then all cohorts are pooled as individual and mean ± SEM curves**. (B)** Experimental design. All included mice were reconstituted with Hu-CD34^+^ cells from three different donors (cohorts 1–3) and harbored >25% Hu-CD45^+^ cells in blood at 12 weeks post-HSC inoculation. They were engrafted with MBA-MB231 breast tumor cell line and then treated with either isotype control antibody (black lines) or anti-PD1 mAb, Pembrolizumab (red lines). **(C)** Tumor growth of individual mice (upper panel) and mean ± SEM (lower panel) are shown for the 3 separated cohorts (cohort 1: n=2-3, cohort 2: n=6, cohort 3: n=5). Global p value was obtained with a Two-Way ANOVA-Type with Box approximation and calculated between untreated/anti-PD1 treated groups for each cohort. Early terminations were due to premature death of the animals, or to humane endpoints taking into consideration the health status of the mice; but not to GvHD. These experiments were performed at Crown Bioscience.

A mini-PDX trial was conducted by injecting seven PDL-1 high small-cell lung carcinoma (SCLC) PDXs in NSG mice humanized with Hu-CD34+ cells derived from 5 different donors (**Figure 3A**, **Supplementary Figure 3**). We observed that in four out of five cohorts (cohorts 1, 2, 3, and 5), anti-PD1 mAb treatment led to tumor-growth inhibition in at least one PDX model tested. However, in cohort 4, no response to anti-PD1 therapy was detected, suggesting that immunotherapy outcomes in the Hu-CD34+ model can be significantly influenced by the HSC donor, independent of tumor-intrinsic factors.

To further evaluate the tumor-intrinsic sensitivity to anti-PD1 mAb, we pooled the tumor-growth curves for each PDX across all cohorts. As shown in the two bottom rows of **Figure 3A****, SCLC** PDXs #1, 4, 5, and 7 displayed no response to anti-PD1 treatment, whereas PDXs #2, 3, and 6 exhibited only weak partial responses. However, given the overall lack of strong responses and the variability in tumor growth kinetics across cohorts, it remains challenging to draw definitive conclusions regarding the tumor-intrinsic sensitivity to anti-PD1. The mild responses observed in certain PDXs may reflect variability in the dispersion of response curves rather than a consistent tumor-intrinsic susceptibility to therapy.

A similar degree of response heterogeneity was observed in a tumor model using a CDX, the MDA-MB-231 breast tumor cell line (**Figures 3B, C**). While one cohort demonstrated complete responses in all three mice, the other two cohorts exhibited weak to no tumor regression following anti-PD1 treatment.

Collectively, these findings highlight the significant impact of the HSC donor on ICI therapy outcomes and reveal key limitations of the Hu-CD34+ model for preclinical immunotherapy evaluation, particularly in studies with necessarily small cohort sizes, where results should be interpreted as case-study–type observations rather than definitive efficacy assessments.

### 3.4 Graft versus tumor and GvHD in the Hu-PBMC model: a crosstalk between human tumors and immune cells

**Figure 4:**
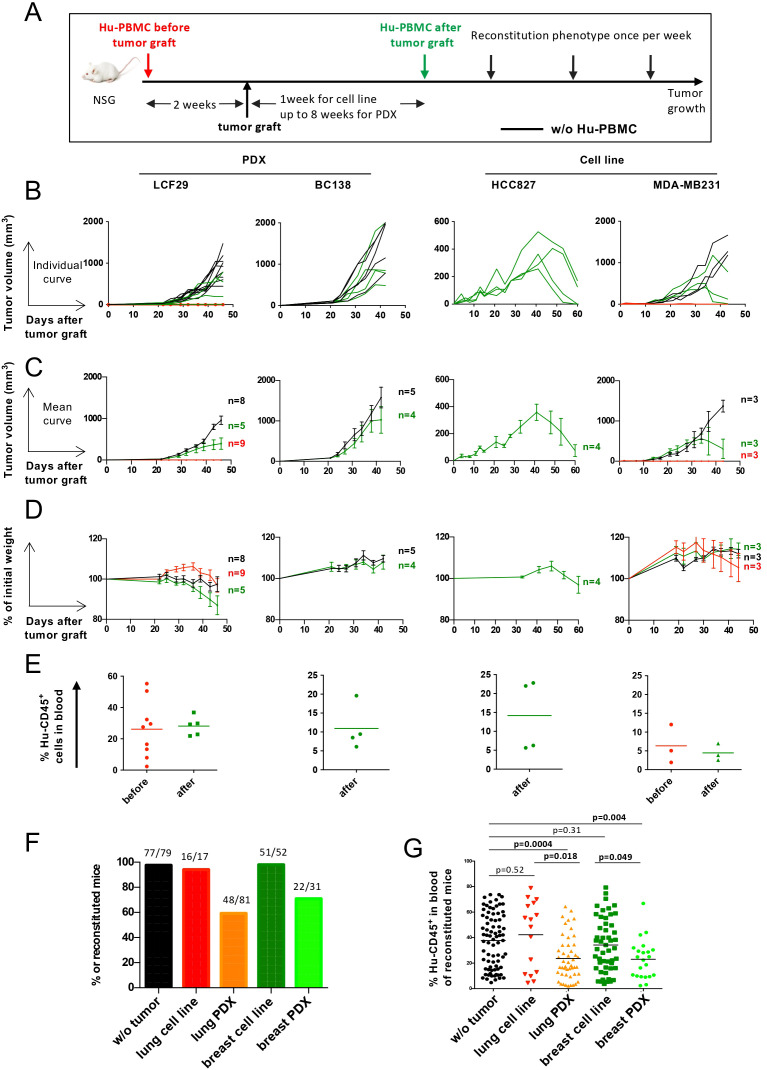
**A** crosstalk between human tumors and human immune cells. **(A)** Experimental design. 5 or 10x10^6^ CD3^+^ T cell-containing Hu-PBMCs were injected two weeks before tumor graft (red lines) or after tumor graft was detectable (green lines); 1 week for tumor cell lines, up to 8 weeks for PDXs. As reference, tumor growth was measured in mice non injected with PBMCs (black lines). **(B)** Individual tumor growth kinetics of LCF29 (NSCLC PDX), BC138 (TNBC PDX), HCC827 (NSCLC cell line) and MDA-MB231 (TNBC cell line). **(C)** Tumor growth kinetics represented as mean ± SEM of individual curves shown in **(B, D)** GvHD development was followed by weight loss, represented by percentage (%) of initial weight. **(C, D)** Data are represented as mean ± SEM of n=3–9 mice per group. **(E)** Blood was recovered to measure Hu-CD45^+^ cells 30 days after Hu-PBMCs injection for LCF29, 22 days for BC138, 27 days for HCC827, and 20 days for MDA-MB231. **(F, G).** NSG mice were not grafted with tumor (black, n=79) or grafted with NSCLC lung tumor cell line (HCC827, red, n=17), NSCLC PDX (orange, n=81), breast tumor cell line (MDA-MB231, dark green, n=52) or breast PDX (light green, n=31). Then mice were injected with 5x10^6^ to 7.5x10^6^ CD3^+^ T cell-containing Hu-PBMCs. Mice were evaluated for Hu-CD45^+^ staining in blood. **(F)** Proportion of reconstituted mice. Mice were considered reconstituted when more than 2% of Hu-CD45^+^ were detected at two different time points. Numbers above bars indicates number of reconstituted mice/total PBMCs injected mice. **(G)** Frequency (%) of Hu-CD45^+^ cells in reconstituted tumor-bearing mice at the maximal percentage of Hu-CD45^+^ cells detected in F (between day 30 and day 40 after tumor graft) with the mean shown for each group. P values were calculated with a Mann Whitney non-parametric t test. Data is from a pool of several experiments. These experiments were performed at Institut Curie.

We next evaluated the impact of timing and sequence of Hu-PBMC and tumor xenograft injections (**Figure 4A**). As shown in **Figures 4B, C**, when PBMCs were injected 15 days prior to tumor engraftment (PDX-LCF29 or MDA-MB-231 cell line) tumor growth was completely abrogated. In contrast, in tumor-only control mice, both models grew as expected. Importantly, no overt GvHD was observed during the first four weeks (**Figure 4D**), and all mice exhibited successful immune reconstitution, as indicated by the presence of circulating Hu-CD45+ cells in the blood (**Figure 4E**). These findings underscore the potent GvT effect exerted by pre-engrafted PBMCs, which effectively prevent tumor growth.

Conversely, when PBMCs were injected after tumors became palpable, all four tested tumor models continued to grow. Notably, three tumors (PDX-LCF29, PDX-BC138, and the MDA-MB-231 cell line) showed slower growth when PBMCs were injected after tumor implantation, coinciding with hCD45^+^ reconstitution and indicating a delayed, yet detectable GvT effect. Although reduced tumor growth in PDX-bearing mice could theoretically stem from residual patient immune cells interacting with donor PBMCs, this is unlikely because (i) the cell line MDA-MB-231, which contains no patient immune cells, also exhibited delayed growth, and (ii) the late-passage PDXs used are highly unlikely to retain original patient immune cells.

For tumors with slower intrinsic growth kinetics (such as HCC827 and MDA-MB-231), prolonged monitoring revealed eventual tumor regression, further supporting a delayed GvT effect in these models. For the more aggressive PDX-BC138 model, analysis at 4 weeks post-engraftment revealed that up to 10% of the tumor infiltrate consisted of Hu-CD45+ immune cells, primarily CD4+ T cells, along with lower proportions of CD8+ T cells and NK cells (**Supplementary Figure 4**).

Interestingly, circulating Hu-CD45+ cell levels, as well as the kinetics and intensity of GvHD, varied across tumor models and did not predict tumor growth control (**Figures 4D, E**). The mechanisms underlying GvHD onset in this model may involve the mismatch of major histocompatibility complex (MHC) molecules between the engrafted human immune cells and the mouse host tissue (xeno-GvHD), as well as the allo-response of T cells from the donor recognizing and attacking different human MHC molecules present in the tumor (13, 23). When comparing pooled data from multiple experiments, we observed lower frequencies of Hu-PBMC reconstitution in PDX models than in tumor cell line-engrafted mice or tumor-free controls, suggesting that PDX models may negatively impact PBMC engraftment (**Figure 4F**). Additionally, the maximum PBMC reconstitution levels, typically reached between 30–40 days post-injection, varied across conditions (**Figure 4G**). Despite high inter-mouse variability in Hu-CD45+ percentages, tumor-free and tumor cell line-engrafted mice generally exhibited superior PBMC reconstitution compared to PDX-bearing mice, reinforcing the notion that tumor type can influence immune cell dynamics.

Overall, these findings highlight a bidirectional interplay between engrafted human immune cells and tumors. While PBMCs can mediate a robust GvT effect against certain tumors, the presence of PDX tumors can, in turn, hinder immune cell reconstitution. Consequently, to standardize the Hu-PBMC tumor model for subsequent studies, we established a protocol wherein tumors were always engrafted prior to PBMC injection, with concurrent monitoring of immune reconstitution and mouse body weight to account for the above-mentioned variables.

### 3.5 Pre-clinical evaluation of anti-PD1 treatment in the Hu-PBMC model

**Figure 5:**
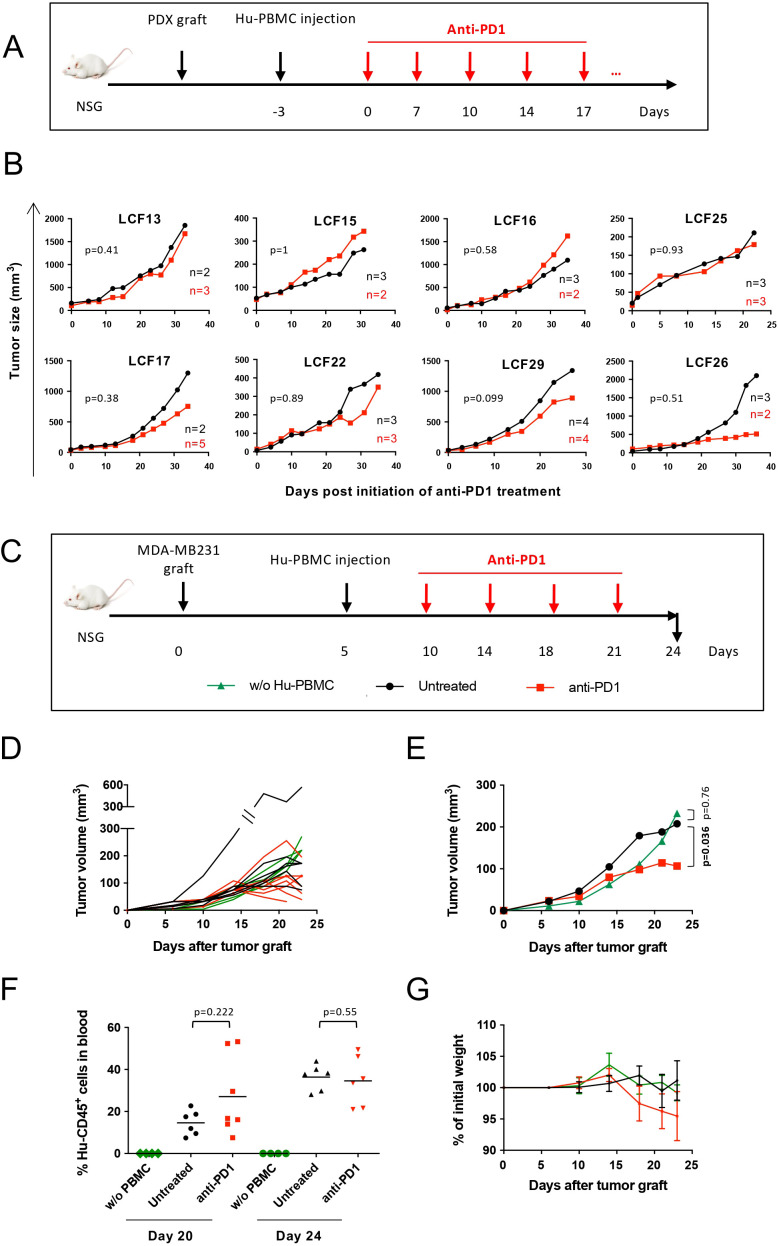
Effect of anti-PD1 treatment on tumor growth in Hu-PBMC-NSG model. **(A)** Experimental design. NSCLC PDX-bearing NSG mice were injected with 5 to 7.5x10^6^ CD3^+^ T cell-containing Hu-PBMCs and treated 3 days after Hu-PBMCs injection either with anti-PD1 (Nivolumab) twice per week (red line, n=2-5) or with PBS (black line, n=2-4). To ensure consistency, only mice showing > 2% Hu-CD45+ cells in circulation at least at two different time points during the experiment were included in the control and treatment groups for analysis. Mice were then treated and there were cases where tumors failed to develop either in control anti- PD-1 groups. **(B)** Tumor growth kinetics with 8 NSCLC PDX models. Data is represented as mean of the tumor size. **(C)** Experimental design. NSG mice were grafted with MDA-MB231 breast tumor cells. Once tumor was detectable (tumor size range= 10–30 mm^3^), mice were injected with 5x10^6^ CD3^+^ T cell-containing Hu-PBMCs. Five days after Hu-PBMCs injection, mice received PBS (black, n=5) or anti-PD1 mAb (Nivolumab) (red, n=7) twice per week. Control mice did not receive Hu-PBMCs (green, n=4). **(D)** Tumor growth kinetic of one representative experiment out of two, shown as individual curves. **(E)** Tumor growth kinetic shown as the mean from mice shown in the figure 5D. Global p value was obtained with a Two-Way ANOVA-Type and p<0.05 are considered as significant. **(F)** Frequency (%) of Hu-CD45^+^ cells in blood at day 20 and day 24. P values were calculated with non-parametric t test on log-transformed data **(G)** GvHD development was followed by weight loss. Data is represented as mean ± SEM. No significant differences were obtained in multiple comparison test. These experiments were performed at Institut Curie.

To assess the suitability of the Hu-PBMC model for immunotherapy studies, we evaluated the response of Hu-PBMC NSG mice engrafted with eight different NSCLC-PDXs to anti-PD1 mAb treatment (**Figure 5A**). Four PDX models (LCF13, LCF15, LCF16, and LCF25) showed no detectable response to anti-PD1 therapy, whereas the remaining four models (LCF17, LCF22, LCF29, and LCF26) displayed only mild trends toward response that did not reach statistical significance (**Figure 5B****).**

Immune reconstitution varied significantly across PDX-bearing mice, and anti-PD1 mAb treatment correlated with reduced PBMC engraftment in 6 of the 8 models tested (**Supplementary Figure 5A****).** Using the MDA-MB-231 model, we observed a modest delay in tumor growth following anti-PD1 therapy compared to controls (**Figures 5C–E**). At day 20 post-PBMC injection, anti-PD1-treated mice exhibited variable levels of circulating Hu-CD45+ cells, with no statistically significant difference compared to untreated mice (**Figure 5F**), reflecting the heterogeneity of immune reconstitution in this model, and coinciding with the onset of weight loss (**Figure 5G**). Notably, weight loss in anti-PD1-treated mice may reflect treatment-related toxicity rather than purely an anti-tumor effect. At the experimental endpoint, both untreated and anti-PD1-treated tumors exhibited high levels of Hu-CD45+ immune cell infiltration (**Supplementary Figure 5B**), with no discernible differences between treatment groups.

Although tumor control by anti-PD1 therapy was mild and heterogeneous, similar findings were independently reproduced in a separate laboratory (**Supplementary Figures 5C–F**), demonstrating the reproducibility of these results. However, the observed responses underscore the limited efficacy of anti-PD1 mAb in Hu-PBMC models, likely due to their unique immune environment.

### 3.6 Pre-clinical evaluation of other anti-tumor T-cell directed therapies in the Hu-PBMC model

Given the limited efficacy of anti-PD1 mAb observed in our previous experiments and the heterogeneous responses across different tumor models, we sought to explore other immunotherapies that may better harness T-cell-mediated tumor rejection. We focused on two additional promising T-cell-directed therapies: an IL-2-based immunotherapy (IL-2Cx) (24) and a T-cell engager (EpCAM-CD3 bispecific antibody (25). Both therapies have shown potential in enhancing anti-tumor immunity, particularly by promoting the activation of specific T-cell subsets, which are critical for mounting effective anti-tumor responses. Moreover, these therapies offer alternative mechanisms of immune activation that could overcome some of the limitations observed with PD-1 blockade.

**Figure 6:**
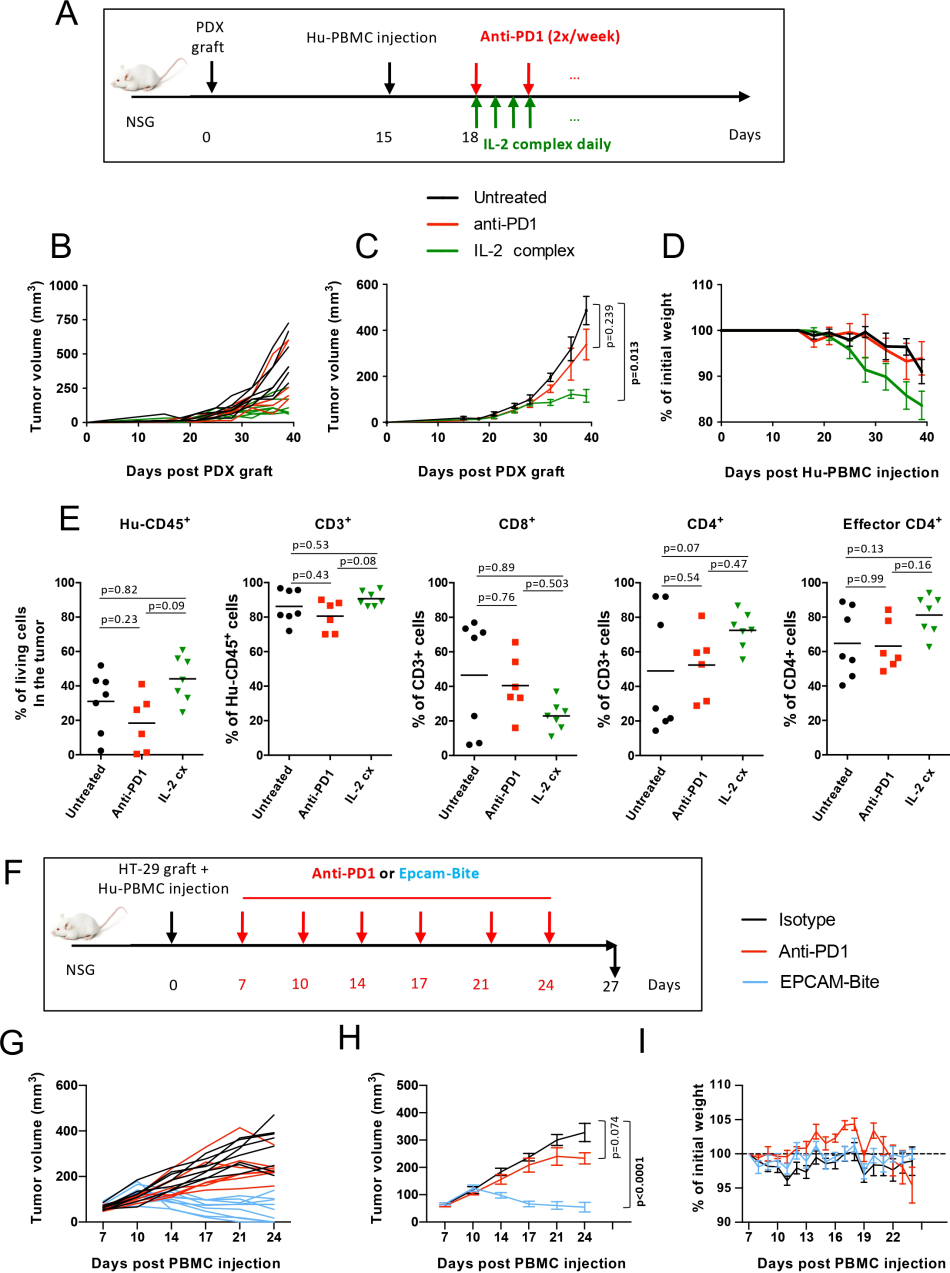
T-cell driven therapies show high anti-tumor efficacy in the Hu-PBMC model. **(A)** Experimental design of experiment performed at Institut Curie. NSCLC LCF29 PDX-bearing NSG mice were injected with 7.5x10^6^ CD3^+^ T cell-containing Hu-PBMCs. Mice were treated either with PBS (black, n=7), anti-PD1 mAb (Nivolumab) (red, n=6), or IL-2Cx (green, n=7). Only reconstituted mice were included (i.e., mice showing > 2% Hu-CD45^+^ cells in circulation in at least two different time points during the experiment). **(B, C)** Tumor growth kinetics represented as individual curves **(B)** and as mean ± SEM of n=6–7 mice per group **(C, D)** GvHD onset was followed by weight loss represented as mean ± SEM. **(E)** Flow cytometry analysis of tumor infiltrating cells was done 40 days after PDX engraftment (25 days after Hu-PBMCs injection). Frequencies (%) of the indicated human immune cell populations are shown and p values were calculated with One-Way Analysis of Variance on log transformed data and are adjusted with Tukey’s correction for multiplicity. **(F)** Experimental design of this experiment performed at Sanofi. NSG mice were injected on day 0 with HT-29 tumor cells and with 10x10^6^ Hu-PBMCs. On day 7, when tumor reached 45–125 mm^3^, mice were treated with anti-PD1 mAb (Nivolumab), or with isotype control or with an EpCAM-CD3. **(G)** Individual HT-29 tumor growth kinetics. **(H)** Tumor growth kinetics represented as a mean ± SEM of n=7–9 mice per group. **(I)** GvHD development was followed by weight loss. Data are represented as mean ± SEM of n=7–9 mice per group. No significant differences were obtained in multiple comparison test. **(C, H)** Global p value was obtained with a Two-Way ANOVA-Type with Bonferroni-Holm’s correction for multiplicity and p<0.05 were considered as significant.

We first evaluated the IL-2/anti-IL-2 antibody complex (IL-2Cx), which preferentially activates NK and CD8+ T cells, key players in tumor elimination. In pre-clinical models of transplantable tumors, IL-2Cx has demonstrated significant efficacy (17, 26). However, its potential had not been previously tested in humanized cancer models. In the LCF29 PDX-bearing Hu-PBMC model, anti-PD1 therapy had no significant impact on tumor growth, whereas IL-2Cx treatment induced marked tumor inhibition (**Figures 6A–C**). However, this anti-tumor activity was accompanied by accelerated GvHD development, as evidenced by increased body weight loss (**Figure 6D**). Flow cytometry analysis revealed that IL-2Cx-treated mice exhibited an increased proportion of CD4+ T cells relative to CD8+ T cells in circulation (**Supplementary Figure 6**). In some mice, a sizeable proportion of these IL-2Cx-expanded CD4+ T cells displayed an effector phenotype, and importantly, CD4+ CD25+ T cells did not express FoxP3 (data not shown), indicating that IL-2Cx primarily activated conventional (non-Treg) CD4+ T cells in this setting. At 40 days post-PDX engraftment, immune-cell infiltration patterns in IL-2Cx-treated tumors were heterogeneous, and no statistically significant differences in Hu-CD45^+^, CD3^+^, CD8^+^, CD4^+^, or effector CD4^+^ T-cell frequencies were observed among groups (**Figure 6E****).**

Next, we tested the EpCAM-CD3 bispecific antibody, which engages T cells through direct TCR activation (anti-CD3 Ab arm) and targets tumors via EpCAM recognition on the surface of tumor cells. As shown in **Figures 6F–H**, anti-PD1 mAb induced a slight tumor growth delay, whereas EpCAM-CD3 treatment led to significant tumor regression, with complete tumor resolution observed in 3 out of 9 mice (**Figures 6F–H**), and no significant differences in body weight (**Figure 6I****).** providing strong evidence for the potential of bispecific antibodies in inducing potent anti-tumor responses.

Overall, these results highlight the utility of the Hu-PBMC model for evaluating immunotherapies, particularly those that directly and robustly activate T cells. Unlike PD-1 blockade, which shows heterogeneous responses likely influenced by donor variability, therapies such as IL-2Cx and EpCAM-CD3 bispecific antibodies induce strong T-cell activation, leading to more consistent and reproducible immune responses in this model.

## 4 Discussion

The development of humanized mouse models has provided an essential platform for studying human immune responses and evaluating immunotherapies *in vivo*. While more sophisticated models have been developed in recent years (5), our study remains one of the few to systematically compare the key variables influencing immune reconstitution and therapy response across the most widely used humanized mouse models.

Consistent with previous studies (18), we confirm that Hu-PBMC and Hu-CD34+ HSC models show different engraftment patterns of human immune cell reconstitution. The Hu-PBMC model rapidly establishes a T-cell-dominated immune environment, within 1–2 weeks, with activated and proliferative CD3+ T effector cells driving immune responses (27)). In contrast, the Hu- CD34+ model requires 10–12 weeks for slow allows for multilineage hematopoietic reconstitution, including myeloid, B, T, and NK cells, but requires prolonged reconstitution time, as also reported by Maser et al. (12).

Additionally, we observed that the sequence of injection of tumor and PBMCs conditions tumor engraftment. We tried to find an optimal condition by varying PBMC injection timing (7, 14, and 21 days before tumor inoculation) and adjusting PBMC doses (5 × 10^6^ vs. 10 × 10^6^ CD3^+^ T cell–containing PBMCs). Across these conditions, early PBMC engraftment consistently led to rapid and complete tumor rejection, even for aggressive models such as TNBC and NSCLC, due to strong GvT reaction. For fast-growing tumors, shortening the interval between PBMC injection and tumor inoculation (≤7 days) was tested but still resulted in significant tumor growth inhibition. These findings indicate that PBMC pre-engraftment creates an immune environment incompatible with tumor establishment rather than a failure of tumor growth per se. Consequently, PBMC pre-tumor designs are not suitable for modeling tumor progression under immune pressure, and PBMC-based HIS models are better suited for post-tumor engraftment protocols when evaluating immunotherapies.

While Hu-CD34+ models theoretically offer a more complete immune landscape, we observed considerable donor-to-donor variability in immune composition and responses to therapy. This heterogeneity aligns with prior reports highlighting the challenges of modeling individualized immune responses in humanized mice (28). Interestingly, we unexpectedly observed that CDX models support better PBMC reconstitution than PDX models, likely due to their simpler, less immunosuppressive tumor microenvironment and potential differences in MHC expression.

Several mechanisms may underlie the observation that tumor type significantly influences immune reconstitution and therapy responsiveness in HIS models. First, patient-derived xenografts (PDXs) often maintain stromal components, and may contain residual patient-derived immune cells, which could influence PBMC engraftment through allo-reactive mechanisms and contribute to variability in immune reconstitution. Additionally, PDX may retain and cytokine networks from the original tumor microenvironment (29), which can modulate immune cell engraftment by promoting immunosuppressive signaling (e.g., TGF-β, IL-10) or altering chemokine gradients that affect T-cell trafficking. In contrast, cell line-derived xenografts (CDXs) typically lack complex stromal architecture and exhibit higher proliferative rates, which may reduce physical and biochemical barriers to immune infiltration. Second, tumor mutational burden and antigen diversity can shape T-cell activation and exhaustion, influencing responsiveness to checkpoint blockade. For example, NSCLC PDXs with high mutational load may provide stronger antigenic stimulation (30), whereas TNBC models often display immunologically ‘cold’ phenotypes with limited antigen presentation (31). Third, donor-dependent variability interacts with tumor-intrinsic factors: PBMCs from different donors exhibit distinct proportions of effector memory T cells and regulatory T cells, which can amplify or dampen GvT effects. Finally, cytokine profiles measured in plasma (e.g., IFN-γ, TNF-α) suggest that the tumor-immune interactions may result in activation and inflammatory signaling, accelerating GvHD onset and limiting therapeutic windows. These hypotheses warrant further investigation through integrated analyses of tumor transcriptomics, immune phenotyping, and functional assays in HIS models.

To study immunotherapies in HIS models, we selected various tumor models (TNBC, SCLC, NSCLC, CRC) because they represent clinically relevant indications for PD-1 blockade and T-cell engaging therapies, and they exhibit distinct immune microenvironments- critical for evaluating HIS model performance. In the context of PD-1 blockade, our findings confirm the limited efficacy of anti-PD-1 monotherapy in Hu-CD34+ models. Despite its clinical success in NSCLC patients (32–34), PD- 1 blockade alone did not elicit strong anti-tumor responses across multiple tumors in our study. This observation mirrors findings from other studies suggesting that Hu-CD34+ models may not fully recapitulate the immunological complexity required for robust checkpoint blockade efficacy (27). One possible explanation is the limited functional maturation of antigen-presenting cells (APCs) in these models, which may impair effective T-cell priming and response to checkpoint inhibitors (8, 35). Additionally, the absence of key human cytokines and growth factors in these mice likely constrains optimal T-cell activation, which is crucial for PD-1 blockade efficacy. The efficacy of anti-PD-1 antibodies could be influenced by immune cells originally infiltrating the grafted PDX, particularly at early passages. Moreover, because the TCR repertoire within 5×10^6^ transplanted PBMCs represents only a small fraction of a patient’s immune system, limited diversity may contribute to inter-mouse variability. In addition, in cases of early passages PDXs with residual T cells, it will be difficult to determine whether PD-1 blockade primarily affects tumor-infiltrating cells or the transplanted PBMCs.

In contrast, our mouse clinical trial using NSCLC PDXs and anti-PD-1 mAb revealed a response rate (~50%) that was reminiscent of clinical response rates in NSCLC patients (21.3% objective response and 39.9% disease control rate) (36). This underscores the importance of immune donor variability and tumor-specific factors in shaping responses to PD-1 blockade. While this suggests that the Hu-CD34+ model may capture some aspects of clinical heterogeneity, it also emphasizes that additional immune components, such as myeloid cells and stromal interactions, need to be optimized in future models to better reflect patient responses. In Hu-CD34^+^ mice, as in Hu-PBMC models, the efficacy of anti-PD-1 mAb may be influenced by immune cells originally present in early-passage PDXs, making it difficult to distinguish effects on tumor-infiltrating cells from those on CD34^+^-derived immune cells.

In contrast to the limited effects observed with PD1-blockade, Hu-PBMC models showed robust responses to T-cell engaging therapies, including IL-2Cx and EpCAM CD3 bispecific antibodies. IL-2Cx demonstrated significant anti-tumor activity, primarily through CD4+ T-cell expansion. While this differs from previous reports in syngeneic mouse models showing predominant CD8+ and NK cell activation (18), it aligns with emerging evidence that IL-2-based therapies can preferentially expand CD4+ subsets in humanized settings (37, 38). Future studies should dissect the relative contributions of CD4+ versus CD8+ T cells to the GvT response induced by IL-2Cx. Similarly, EpCAM-CD3 bispecific antibodies induced robust tumor regressions in the Hu-PBMC model, with complete responses in 3 of 9 mice. This supports prior findings that humanized mice provide a relevant platform for testing bispecific T-cell engagers (TCEs), which rely on direct and potent T-cell activation (39, 40). The ability of Hu-PBMC models to reproducibly capture T-cell activation dynamics suggests they may be particularly useful for evaluating therapies that bypass classical antigen presentation and rely on direct T-cell engagement.

Despite the demonstrated value of both Hu-CD34+ and Hu-PBMC models in immuno- oncology research, our findings highlight the need for further refinement to enhance their translational relevance. The Hu-CD34+ model offers multilineage immune reconstitution, extended study windows, and minimal GvHD, making it suitable for long-term evaluation of checkpoint inhibitors and therapies requiring antigen presentation. However, it is limited by long reconstitution times, high cost, incomplete myeloid maturation, and donor variability. In contrast, the Hu-PBMC model provides rapid T-cell engraftment and strong T-cell activation, enabling robust responses to T-cell–engaging therapies such as IL-2Cx and bispecific antibodies. Its limitations include short therapeutic windows (2–4 weeks) due to inevitable GvHD and a T-cell-dominant immune landscape. Among mouse strains, NSG and NOG represent stable reference models, while NSG-SGM3 and NOG-EXL enhance myeloid differentiation but introduce toxicity risks. Translationally, Hu-PBMC models are ideal for testing T-cell–dependent therapies and cytokine storm risk, whereas Hu-CD34+ models are better suited for checkpoint blockade and mechanistic studies of immune development.

The limitations of HIS models are mainly related to donor variability, incomplete myeloid differentiation, xenogeneic GvHD, and murine stromal components influencing immune-tumor interactions. In this study, PBMC donors were not systematically HLA-matched across tumor models, which may contribute to variability and underscores the importance of incorporating HLA matching in future work. These limitations define the boundaries of interpretation and highlight the need for improved models incorporating human cytokines and stromal components. Along these lines, the development of transgenic humanized mouse strains expressing human cytokines (e.g., IL-15, GM-CSF, IL-3, CSF) represents a promising avenue to enhance immune functionality and better model human immune-tumor interactions (5, 41).

One particularly promising approach is the immune-avatar strategy, in which early-passage PDXs derived from patient tumors are co-engrafted with autologous immune cells to evaluate therapy responses in a personalized manner to avoid mismatched immune cells and tumor (38, 42–44). Given the difficulty of obtaining Hu-CD34+ cells from cancer patients, Hu- PBMC models offer a feasible alternative for implementing this strategy. However, challenges remain, particularly in mitigating the impact of graft-versus-host disease (GvHD) an extending the duration of immune response studies. The generation of MHC I/II knockout models (14, 45)may help address these limitations, enabling more stable long-term studies.

Humanized mouse models are also essential tools for the preclinical evaluation of CAR- T cell therapies, to monitor CAR-T cell expansion, antitumor activity, cytokine release, and donor-specific responses (46, 47). While not the focus for the current studies, humanized mouse models also provide a platform to evaluate potential toxicities such as cytokine release syndrome and off-target effects in a controlled environment before clinical trials for immunotherapies such as T cell engagers and CAR-T cells.

## 5 Conclusion

Overall, these results emphasize that while humanized mice remain indispensable tools for preclinical immuno-oncology research, continued refinement is needed to fully capture the complexity of human immune responses. Emerging transgenic models and patient-derived immune-avatar strategies hold promise for bridging the gap between preclinical testing and clinical translation, paving the way for more precise and effective cancer immunotherapies.

## Data Availability

The original contributions presented in the study are included in the article/**Supplementary Material**. Further inquiries can be directed to the corresponding author.
