# Supplementary material for: A comprehensive analysis of humanized mouse models for the study of cancer immunotherapies
Source: Front Immunol. 2026 Apr 7;17:1730378. doi: 10.3389/fimmu.2026.1730378 (PMC13096022; doi:10.3389/fimmu.2026.1730378)
Supplement: Supplementary file 1 [file DataSheet1.pdf]

Supplementary Figure 1

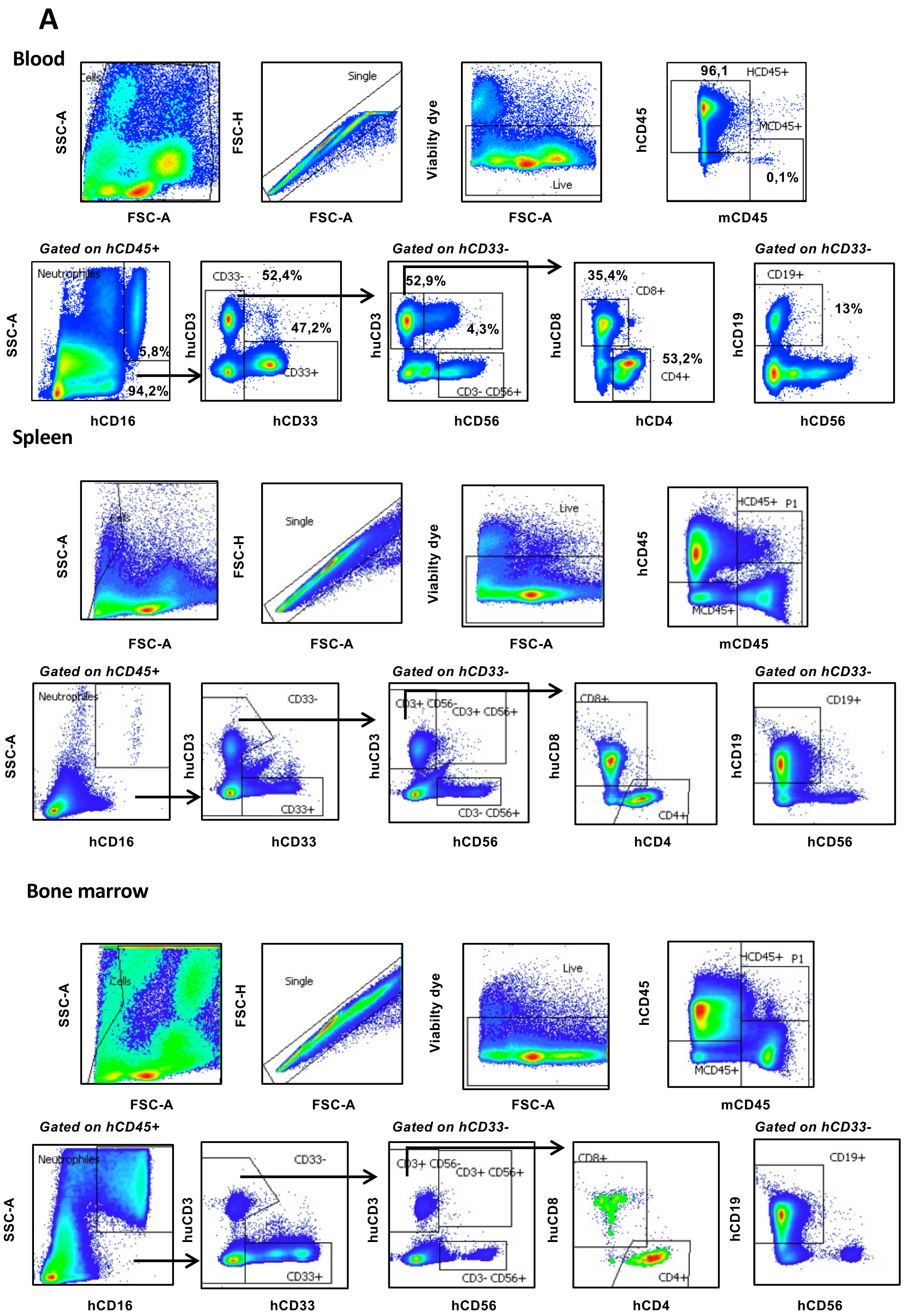

Supplementary Figure 1

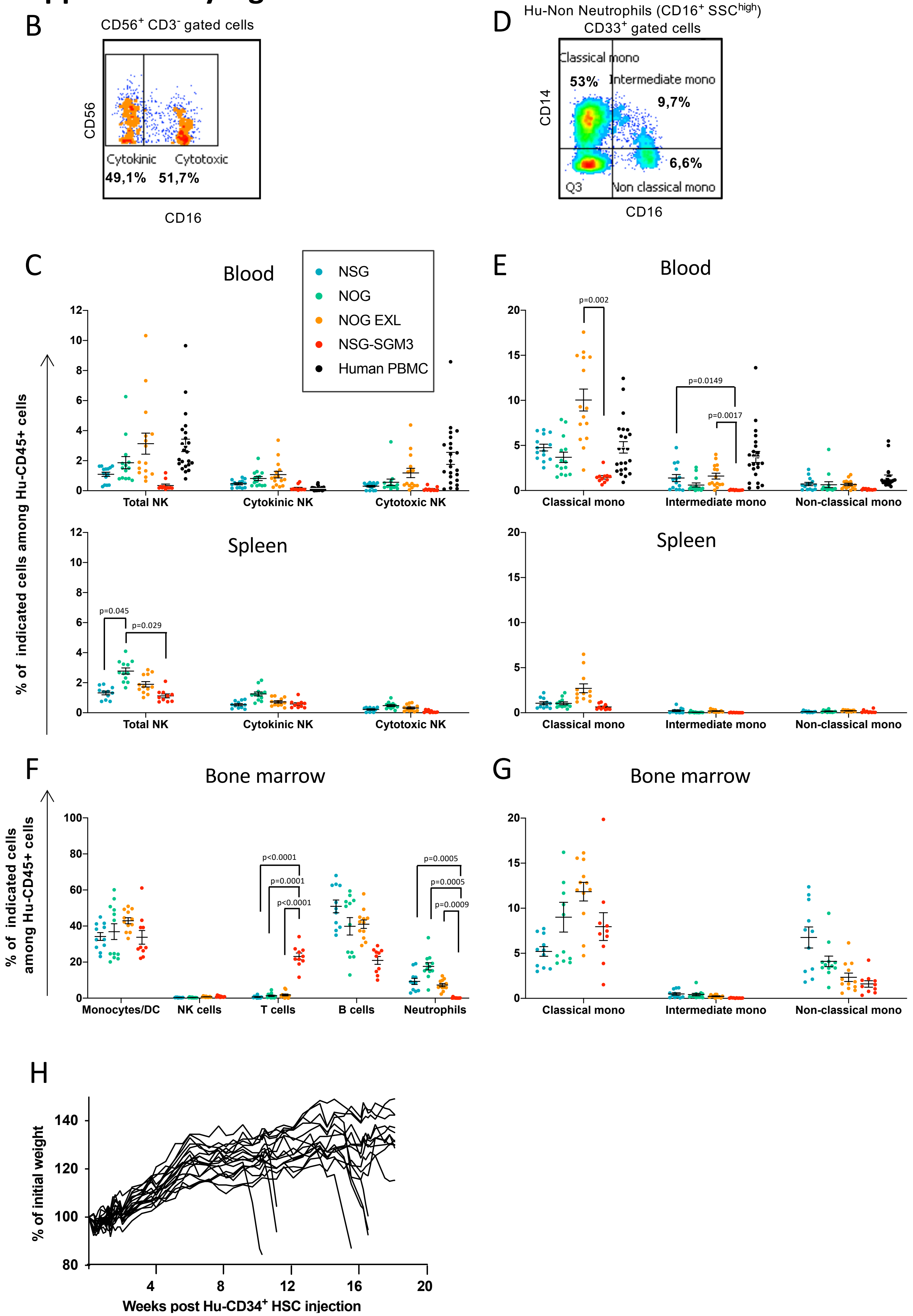

Supplementary Figure 1: Characterization of the NK and Myeloid cell reconstitution upon Hu-CD34$^+$ HSC injection. A. Flow cytometry gating strategy for analysis of human immune cell subpopulations, shown in blood, spleen and bone marrow of Hu-CD34$^+$ HSC-humanized mouse at week 18 after humanization as in Figure 1C. Similar gating strategy was used for Hu-PBMC-NSG mouse in Figure 2. **B.** Flow cytometry gating strategy used to define cytotoxic (CD16$^+$), and cytokinic (CD16$^-$) NK cells among Hu-CD56$^+$CD3$^-$ gated cells (see Supplementary Figure 1A for gating strategy) in blood from Hu-CD34$^+$-humanized mice at week 18. **C.** Percentage of NK cell subpopulations relative to Hu-CD45$^+$ cells in blood and spleen at week 18. **D.** Flow cytometry gating strategy used to define classical (CD14$^+$ CD16$^-$), intermediate (CD14$^+$ CD16$^+$), and non-classical (CD14$^-$ CD16$^+$) monocytes among Hu-Non Neutrophils (CD16$^+$ SSC^{high}) CD33$^+$ gated cells (see Supplementary Figure 1A for gating strategy) in blood from Hu-CD34$^+$-humanized mice at week 18. **E.** Percentage of monocyte subpopulations relative to Hu-CD45$^+$ cells in blood and spleen at week 18. **F.** Distribution of the Hu-CD45$^+$ cell subpopulations relative to Hu-CD45$^+$ cells in bone marrow of different mouse strains at week 18. **G.** Percentage of monocyte subpopulations relative to Hu-CD45$^+$ cells in bone marrow at week 18. Data were obtained from mice reconstituted with 2 or 3 different Hu-CD34+ HSC donors with n=10-15 mice per mouse strain for blood, bone marrow and spleen data. Data is expressed as individual dots and mean ± SD. P value was obtained with a One-Way Analysis of Variance on log-transformed data with Tukey's correction for multiplicity. Absence of reported p-values indicates non-significant results ($p \geq 0.05$). **H.** Hu-CD34$^+$ HSC-humanized NSG-SGM3 mice (as described in Figure 1A-B) were monitored for bodyweight (n=18). Results are shown as percentage of initial weight at the time of HSC inoculation.

Supplementary Figure 2

A

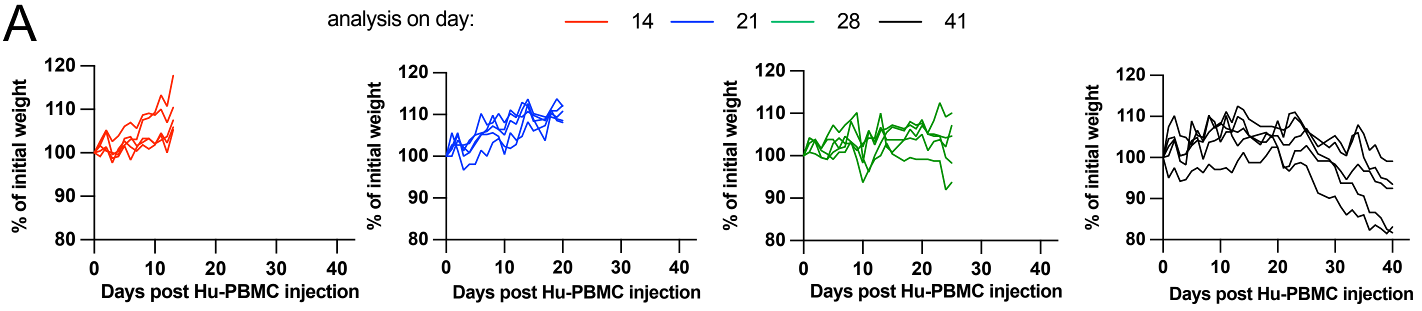

B

Number of injected CD3-containing Hu-PBMC x10^6 (Total injected Hu-PBMCs)	Frequency of reconstituted mice	Mean % of maximum reconstitution	Frequency of GvHD	Mean day of GvHD onset (range)	Mean % (range) of reconstitution at GvHD onset
20-30 (30-50)	2/2 (100%)	49,9%	2/2 (100%)	16 (14-18)	32,7% (20,5-44,8)
10 (16-30)	14/15 (93%)	42,3%	11/14 (78%)	34 (12-51)	38% (15,5-66,3)
5 (9-12)	17/18 (94%)	34,1%	11/17 (65%)	42,3 (29-63)	39,2% (10,7-73,7)
1-3 (2-4)	0/3 (0%)	/	/	/	/

C

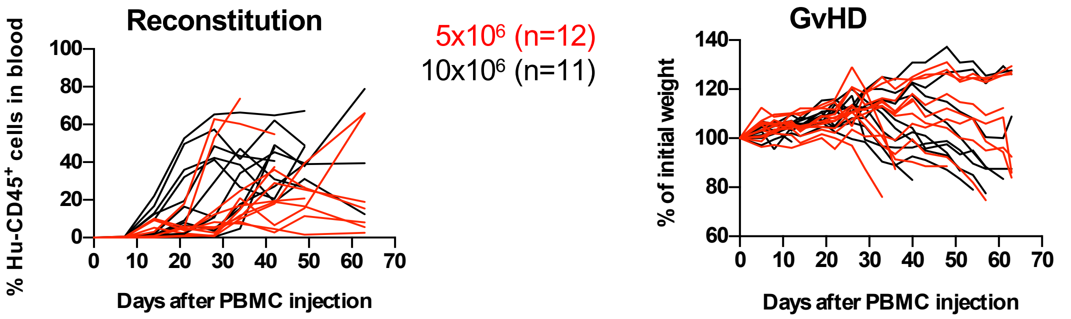

D

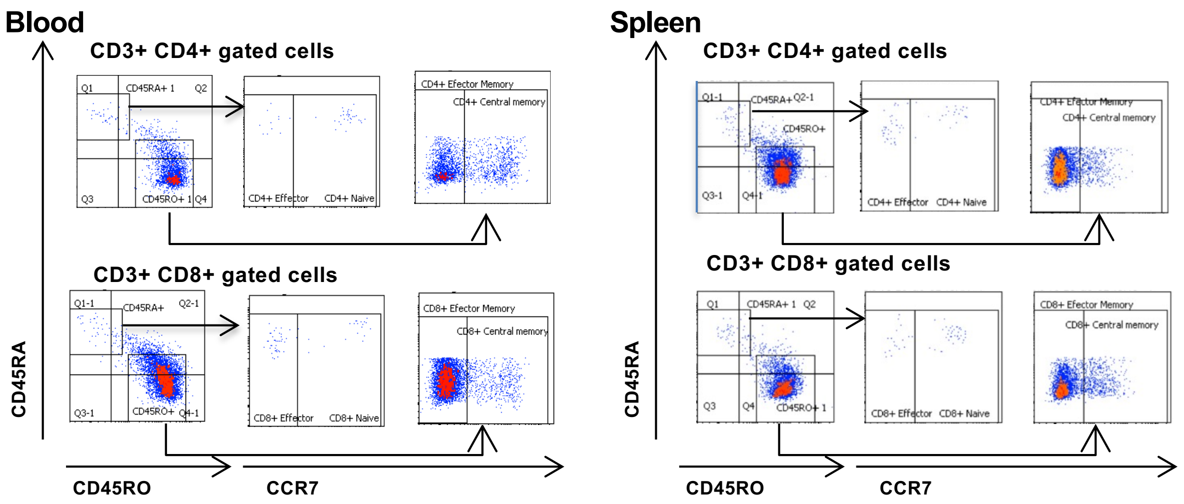

E

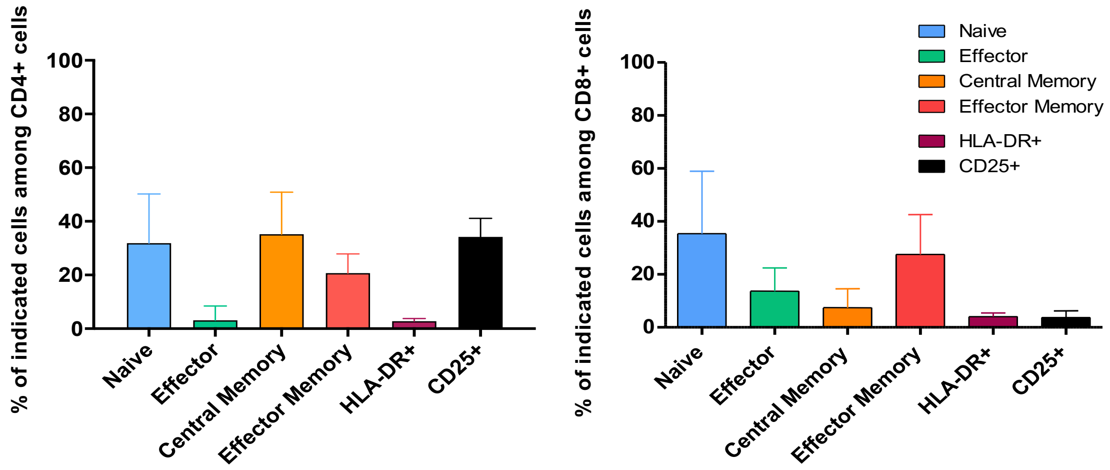

Supplementary Figure 2 : Hu-PBMCs reconstitution and GvHD development in Hu-PBMC-NSG mice
A. For the Hu-PBMC NSG cohort shown in Figures 2A–B, body weight was recorded for five mice at each indicated time point (days 14, 21, 28, and 41) to monitor GvHD onset. **B.** Non-irradiated NSG mice from 8 to 14 weeks of age were injected with different amount of Hu-PBMCs (1 to $30x10^6$) and the Hu-PBMCs engraftment efficiency (measured by the % of Hu-CD45+ cells in blood) and GvHD development (measured by the weight loss) are shown in the table. **C.** NSG mice injected with $5x10^6$ (red lines, n=12) or $10x10^6$ (black lines, n=11) of $CD3^+$ T cell-containing Hu-PBMCs were monitored for reconstitution by quantification of blood circulating Hu-$CD45^+$ (left panel) cells and bodyweight (right panel). Results shown are percentage of initial weight. Statistical analysis on hCD45+ cells was calculated until day 48 with a Two-Way ANOVA on log data for reconstitution and on raw data for GvHD and were not different between the 2 groups. **D.** Flow cytometry gating strategy used to define naïve ($CD45RA^+$ $CCR7^+$), central memory ($CD45RA^-$ $CCR7^+$), effector memory ($CD45RA^-$ $CCR7^-$), and effector cells ($CD45RA^+$ $CCR7^-$) among Hu-$CD4^+$ or Hu-$CD8^+$ gated cells in blood and in spleen from Hu-PBMC-humanized mice at day 14 post-humanization. **E.** T cell differentiation/activation state in blood from healthy donor. Frequencies (%) of naïve, memory, effector memory, and effector cells as well as activation marker-expressing cells (CD25 and HLA-DR) relative to $CD4^+$ or $CD8^+$ T cells of PBMC from healthy donors (n=3-4) used to inject in NSG mice, determined by flow cytometry analysis. Data is expressed as mean ± SD.

Supplementary Figure 3

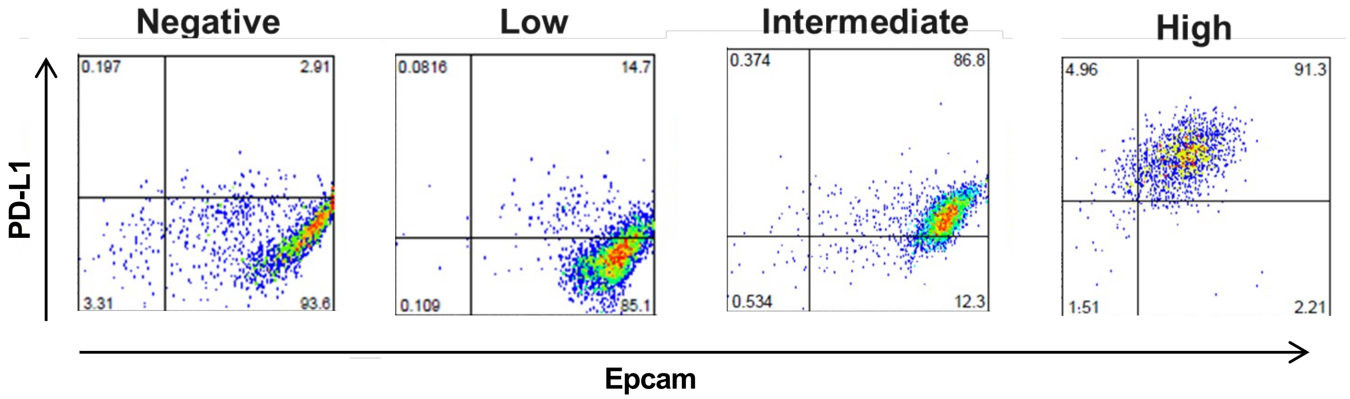

Supplementary Figure 3: PD-L1 expression by SCLC PDXs.
30 SCLC PDX tumors were screened by FACS for their PD-L1 expression level and examples of low / intermediate or high expression are illustrated. 7 high PD-L1 expressers were identified and used in the mini-PDX trial study presented in Figure 3A.

Supplementary Figure 4

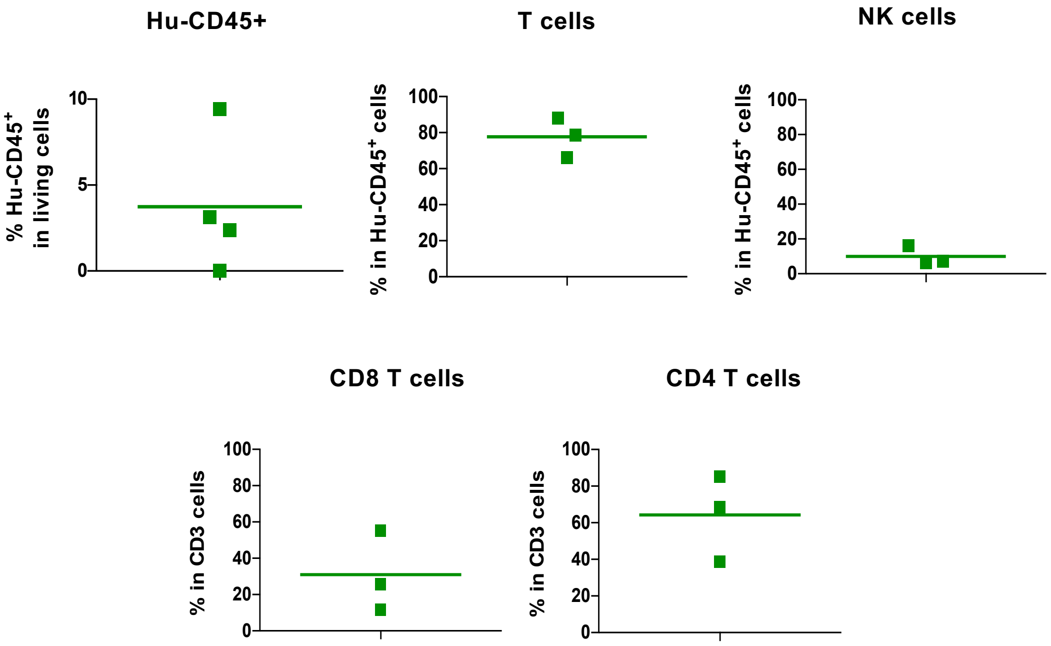

Supplementary Figure 4: Human immune infiltration in TNBC PDX

TNBC PDX (BC138) was sampled 28 days after PBMC injection. Tumor infiltration of human immune cells (Hu-CD45+) was assessed by flow cytometry. Frequencies (%) of tumor infiltrating CD56 (NK cells), CD3 (T cells), CD8 (T cytotoxic cells) and CD4 (T helper cells) are individually shown.

Supplementary Figure 5

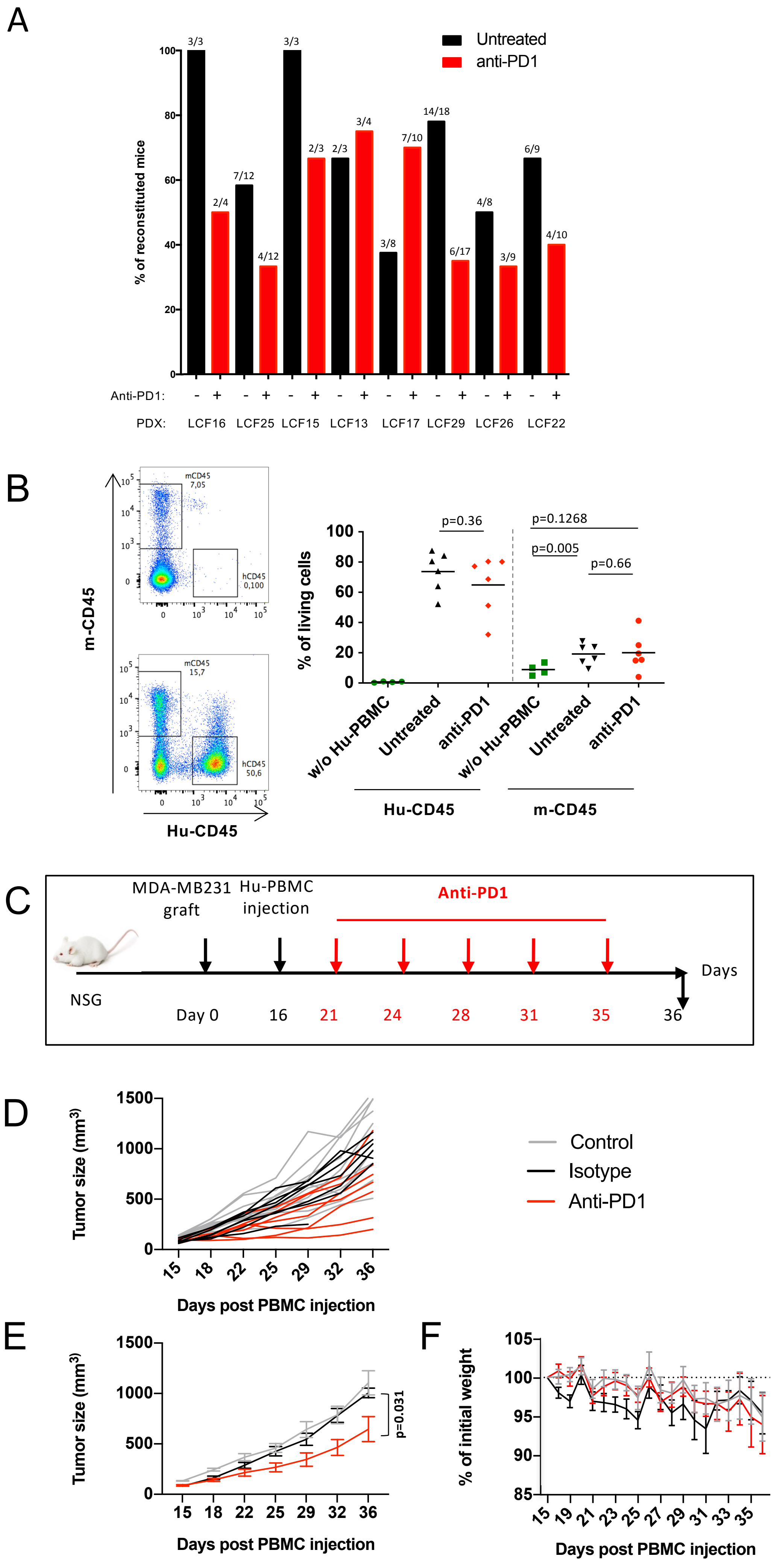

Supplementary Figure 5: Effect of anti-PD1 treatment on lung PDXs or on MDA-MB231 tumor growth in a Hu-PBMC-NSG model.

A. Details of the proportion of reconstituted tumor-bearing mice shown in Figure 5A/B, in 8 NSCLC PDX bearing mice models treated or not with anti-PD1. Mice were considered reconstituted when more than 2% of Hu-CD45$^+$ were detected in blood. Numbers indicated the reconstituted mice/total Hu-PBMCs injected mice. **B.** Mice from the experiment described in Figure 5C were analyzed for the content in Hu-CD45+ or m-CD45+ cells at the tumor site. Representative dot plot showing the frequency (%) of tumor infiltrating m-CD45$^+$ and Hu-CD45$^+$ cells in non-PBMC injected mouse (upper panel, left) and in PBMC-injected mouse (lower panel, left). Quantification of m-CD45$^+$ and Hu-CD45$^+$ cells in tumors from the 3 different groups (without injected PBMC, receiving PBMC and non-treated or anti-PD1 treated) is shown on the right panel. P values were calculated with non-parametric t test on log-transformed data **C.** Scheme of the experimental design. NSG mice were injected with MDA-MB231 breast tumor cells and on day 16 mice were injected ip with $10x10^6$ Hu-PBMC. On day 21, when tumor reached 61-144 mm^3, mice were treated bi-weekly with 10 mg/Kg of anti-PD1 (Nivolumab) or 10mL/kg of Isotype anti-HEL huIgG4 and the control group was not treated. **D**. Individual MDA-MB231 tumor growth kinetics and **E** Tumor growth kinetics represented as a mean ± SEM of the individual curves shown in (D). Global p value was calculated for the comparison between Isotype Control and anti-PD1 treated group and obtained with a Two-Way ANOVA-Type. **F**. GvHD development was followed by weight loss, represented by percentage (%) of initial weight when treatments started. Data are represented as mean ± SD of n=6-10 mice per group. No significant differences were obtained in multiple comparison test.

Supplementary Figure 6

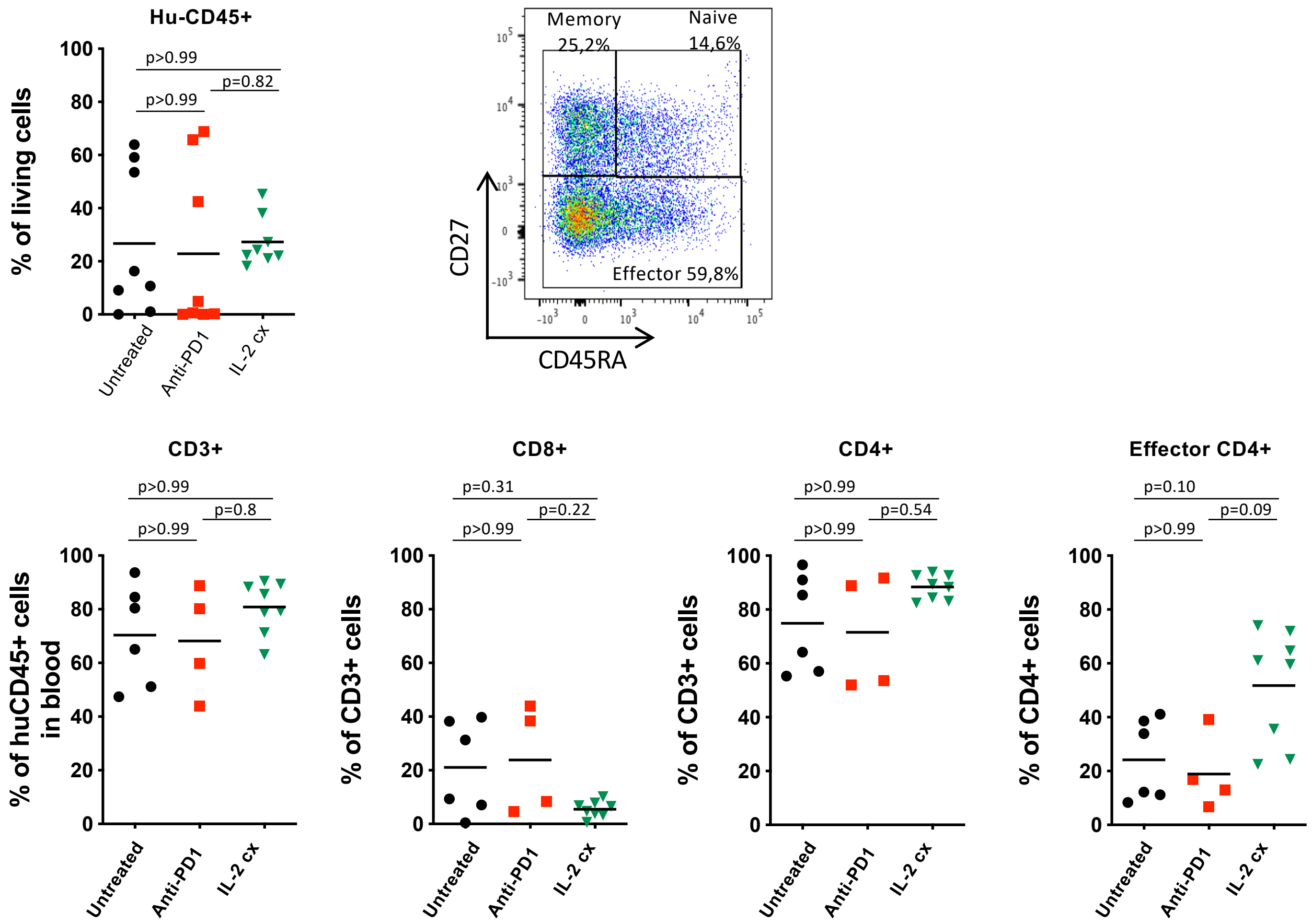

Supplementary Figure 6: IL-2Cx effect on human immune cell reconstitution in the blood of NSCLC-PDX LCF29 Hu-PBMC NSG mice.

LCF29 PDX-bearing NSG mice were treated as described in Figure 6A. Percentages of hCD45+ cells were analyzed in the blood at day 21 after Hu-PBMCs injection (upper panel). Immune cell populations were then quantified after excluding mice without hCD45+ cells (lower panels). Insert illustrates gating strategy to define effector CD4 T cells. P values were calculated with One-Way Analysis of Variance on raw data and are adjusted with Dunn's correction for multiplicity.

Table S1: Antibodies and reagents used for Flow Cytometry.
(The listed antibodies recognize human markers, except the anti-mouse CD45).

Marker	Fluorochrome	Clone	Provider
CD3	BV510	UCHT1	BD
CD3	BV650	OKT3	Biolegend
CD4	BUV395	SK3	BD
CD4	BV785	OKT4	Biolegend
CD8	BUV496	RPA-T8	BD
CD8	PECF594	RPA-T8	BD
CD14	AF700	M5E2	BD
CD16	FITC	3G8	BD
CD19	Alexa 700	HIB19	BD
CD19	BV650	SJ25C1	BD
CD25	PE	M-A251	Biolegend
CD25	BV786	BC96	Biolegend
CD27	BV605	O323	Biolegend
CD33	PECF594	WM53	BD
CD45	BUV805	HI30	BD
CD45	APC Cy7	2D1	BD
CD45RA	BV421	HI100	BD
CD45RA	PECy5	HI100	eBiosciences
CD45RO	APC	UCHL1	BD
CD56	BV421	NCAM16.2	BD
CD56	PE-Cy5	N901	BD
CD197	PECF594	150503	BD
HLA-DR	FITC	AC122	Miltenyi Biotec
PD-1	BV711	EH12.2H7	Biolegend
TCRgd	FITC	11F2	BD
Mouse CD45	PECy7	30-F11	BD
LIVE/DEAD Fixable Aqua	For 405nm Excitation		Life Technologies
Viability Dye	eFluor 780		eBiosciences
